# Parvalbumin Interneuron‐Dependent Hippocampal Neurogenesis Evoked by Prolonged Rhythmic Light Flicker

**DOI:** 10.1002/advs.202503017

**Published:** 2025-06-30

**Authors:** Hai Yan, Yunxuan Wang, Xufan Deng, Shiyu Wu, Yifan Pan, Jun Du, Mei Yu, Bo Liu, Huimei Wang, Zhengyu Zhang, Jinghong Chen, Shuifa Chen, Yizheng Wang, Tara Walker, Perry Bartlett, Jun Ju, Sheng‐Tao Hou

**Affiliations:** ^1^ Brain Research Centre, Department of Neurobiology, School of Life Sciences Southern University of Science and Technology 1088 Xueyuan Blvd Shenzhen Guangdong Province 518055 P. R. China; ^2^ The Brain Science Centre Beijing Institute of Basic Medical Sciences Beijing 100850 P. R. China; ^3^ Huashan Hospital Fudan University 12 Wulumuqi Rd (M), Jing'An Shanghai 200031 P. R. China; ^4^ Clem Jones Centre for Ageing Dementia Research, Queensland Brain Institute The University of Queensland Brisbane QLD 4072 Australia; ^5^ Present address: University of Tübingen Geschwister‐Scholl‐Platz 72074 Tübingen Germany; ^6^ Present address: Shenzhen Bay Laboratory Shenzhen Guangdong 518107 China; ^7^ Present address: Department of Anatomy and Neurobiology, College of Medicine Northeast Ohio Medical University Rootstown USA

**Keywords:** 40 Hz, gamma frequency, adult neurogenesis, rhythmic light flicker, spatial learning

## Abstract

Rhythmic light flicker alleviates cognitive impairments in various animal models of neurological diseases. However, its long‐term effects and underlying mechanisms remain unclear. Here, a cohort of adult mice is subjected to long‐term exposure to 40 Hz light flicker (1 hour daily for 30 days) and observed significant enhancements in hippocampal neurogenesis and spatial learning without any adverse behavioral effects. Specific ablation of hippocampal newborn neurons using DCX^DTR^ mice abolished these effects. Furthermore, the inactivation or elimination of GABAergic parvalbumin (PV) interneurons not only impaired 40 Hz light flicker entrainment but also reduce neurogenesis in the dentate gyrus (DG). Long‐term flicker exposure increases excitatory input to DG PV interneurons, which enhances PV interneuron excitability, elevated GABA levels, and strengthened inhibitory transmission to newborn neurons, thereby promoting better integration of new neurons into the DG. Blocking GABA_A_ receptors reverse the light flicker‐induced increase in neurogenesis and spatial learning. Prolonged flicker exposure do not affect DG regional cerebral blood flow or the activity of excitatory cholinergic, vasoactive intestinal peptide (VIP), or cholecystokinin (CCK) interneurons. These findings suggest that long‐term light flicker enhances spatial learning through PV‐dependent neurogenesis, with elevated GABAergic activity supporting the development and integration of immature neurons in the adult DG.

## Graphical abstract

This study demonstrates that prolonged rhythmic 40 Hz light flicker enhances spatial learning by stimulating PV interneuron‐dependent neurogenesis. The light flicker selectively activates PV interneurons, which enhances GABAergic support for the integration of immature neurons in the adult dentate gyrus. These mechanistic findings provide strong support for developing this non‐invasive sensory stimulation as a therapeutic for treating cognitive impairments.

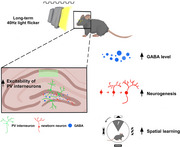

## 1 Introduction

Devastating neurological diseases, such as Alzheimer's disease (AD) and stroke, have a profound impact on human quality of life. In particular, the decline in cognitive functions associated with these conditions significantly impairs individuals.^[^ ^1^, ^2^ ^]^ Currently, therapeutic options for these conditions are limited, representing a significant unmet medical need.^[^ ^3^, ^4^, ^5^ ^]^ As a result, non‐invasive approaches, such as rhythmic light stimulation, are in high demand due to their substantial potential as effective alternatives for mitigating cognitive impairment in both AD and stroke. Recent studies, including our own, have demonstrated significant beneficial effects of rhythmic 40 Hz light flicker in alleviating cognitive deficits in various neurological disease animal models, including those for AD,^[^ ^6^, ^7^, ^8^ ^]^ cerebral ischemia,^[^ ^9^ ^]^ autism spectrum disorders (ASD)^[^ ^10^ ^]^ and chemobrain.^[^ ^11^ ^]^ Importantly, our electroencephalogram studies in human brains have confirmed an increase in 40 Hz entrainment across various brain regions, along with significant alterations in microstates associated with brain functions impaired in AD and stroke patients.^[^ ^12^ ^]^ In fact, the 40 Hz light flicker has received expedited FDA approval and has shown promising results in Phase II clinical trials involving AD patients.^[^ ^13^ ^]^

However, previous studies utilizing light flicker have generally been of short duration, typically lasting less than 14 days. The long‐term effects on cognitive function and the underlying mechanisms remain poorly understood. In this context, we systematically administered long‐term 40 Hz light flicker (1 hour daily for 30 days) to adult mice to assess its impact on cognitive function and the associated mechanisms. We hypothesize that long‐term 40 Hz light stimulation will not induce adverse behavioral effects, but rather enhance cognitive functions by promoting hippocampal neurogenesis through modulation of the interactions between parvalbumin (PV) interneurons and maturing neural cells.

Although, the effectiveness and specific underlying mechanisms of light stimulation remain subjects of ongoing debate,^[^ ^14^ ^]^ several potential mechanisms have been demonstrated, including the enhancement of hippocampal synaptic strength,^[^ ^9^ ^]^ promotion of glymphatic contraction for the clearance of toxic materials,^[^ ^7^ ^]^ activation of microglia,^[^ ^6^ ^]^ modulation of the circadian clock,^[^ ^15^ ^]^ and the activation of specific neurochemical mediators, such as adenosine.^[^ ^10^, ^16^ ^]^

Another critical mechanism for enhancing cognition in the adult brain is neurogenesis, which has not been thoroughly explored in the context of light stimulation. Adult neurogenesis is particularly important for achieving long‐term cognitive improvements in the treatment of neurological diseases such as AD^[^ ^17^, ^18^, ^19^, ^20^ ^]^ and stroke.^[^ ^21^, ^22^, ^23^ ^]^ Mouse brain adult neurogenesis, occurring primarily in the hippocampal dentate gyrus (DG) and the subventricular zone (SVZ) of the lateral ventricles, plays a critical role in learning and memory formation.^[^ ^24^, ^25^, ^26^, ^27^, ^28^ ^]^ These newborn neurons in the DG migrate from the stem cell niche located in the subgranular layer to the granular cell layer, where, after undergoing a limited number of divisions, they integrate into existing neural circuits. However, neurogenesis in the DG declines with age during adulthood.^[^ ^29^, ^30^ ^]^ In contrast, newborn neurons originating from the SVZ migrate along the outer wall of the lateral ventricles toward the olfactory bulb, where they differentiate into olfactory inhibitory interneurons.^[^ ^31^ ^]^

In the DG, specific markers identify distinct stages of neurogenesis. For example, the expression of SOX2 is characteristic of non‐radial and horizontal type‐1 cells. T‐brain 2 (Tbr2) is expressed in type‐2a and type‐2b intermediate neural progenitor cells, which ultimately mature into neuroblasts (type‐3).^[^ ^32^, ^33^, ^34^, ^35^, ^36^ ^]^ Doublecortin (DCX) serves as an early marker in immature granule cells during their migration and dendritic growth; however, its expression is downregulated before these neurons reach maturity. Following this period, the immature neurons establish synaptic connections with existing mature neurons and gradually integrate into the neural circuit.^[^ ^37^, ^38^ ^]^

The precise regulation of neurogenesis in the hippocampus is highly dependent on localized neural circuits that integrate individual experiences, neural activity, and various regulatory mechanisms of neurogenesis.^[^ ^39^, ^40^, ^41^ ^]^ Within these circuits, two primary types of GABAergic interneurons are crucial for supporting the development of newborn neurons: fast‐spiking parvalbumin (PV) and somatostatin‐expressing (SST) interneurons.^[^ ^42^, ^43^ ^]^ PV interneurons are typically located closer to the granule cell layer and provide lateral and recurrent inhibition through continuous GABA release.^[^ ^41^, ^42^, ^43^ ^]^ GABA effectively elevates the resting potential of newborn neurons, primarily due to the high expression of NKCC1 chloride channels.^[^ ^44^, ^45^, ^46^, ^47^ ^]^ This results in a depolarized equilibrium potential for chloride ions in newborn neurons, especially during the first three weeks postnatally.^[^ ^48^, ^49^, ^50^ ^]^ Synaptic connections between PV interneurons and newborn neurons can be observed as early as 7 days postnatally, preceding the connections formed by SST interneurons.^[^ ^48^ ^]^ Optogenetic inhibition of PV interneurons, rather than SST interneurons, effectively suppresses neurogenesis in the DG region.^[^ ^41^ ^]^

Mossy cells are glutamatergic neurons located in the hilus of the DG and are known to exhibit a strong response to spatial exploration.^[^ ^51^, ^52^ ^]^ Although mossy cells provide the first glutamatergic input to adult‐born granule cells,^[^ ^53^ ^]^ deletion of mossy cells does not affect adult neurogenesis.^[^ ^54^, ^55^ ^]^ Other hippocampal interneurons, such as those expressing VIP and CCK, have been recognized for their crucial roles in modulating cognitive functions.^[^ ^56^, ^57^ ^]^ However, their specific involvement in long‐term rhythmic visual stimulation remains unclear.

This study demonstrates that long‐term 40 Hz light flicker entrainment of the hippocampus promotes neurogenesis in the DG and enhances spatial learning. The activation of PV interneurons and the subsequent increase in GABA release are critical in facilitating 40 Hz light flicker‐induced adult neurogenesis and the integration of newborn neurons within the DG. This mechanism provides a novel framework for understanding how light flicker stimulation enhances cognitive function.

## 2 Results

### 2.1 Long‐Term 40 Hz Light Flicker Stimulation Significantly Enhances Spatial Learning

To evaluate the impact of 40 Hz light flicker on cognitive function, 6‐month‐old mice were exposed to daily 1‐hour 40 Hz light flicker sessions for 30 consecutive days. An age‐matched control group was exposed to continuous room lighting without the 40 Hz intervention, serving as a negative control. All mice were housed under a consistent 12‐hour light/dark cycle, with lights on from 7 a.m. to 7 p.m. The 40 Hz light stimulation was administered daily from 6 to 7 p.m. throughout the experimental period, ensuring minimal disruption to the mice's circadian rhythms, as detailed in the Experimental Section.

**Figure 1:**
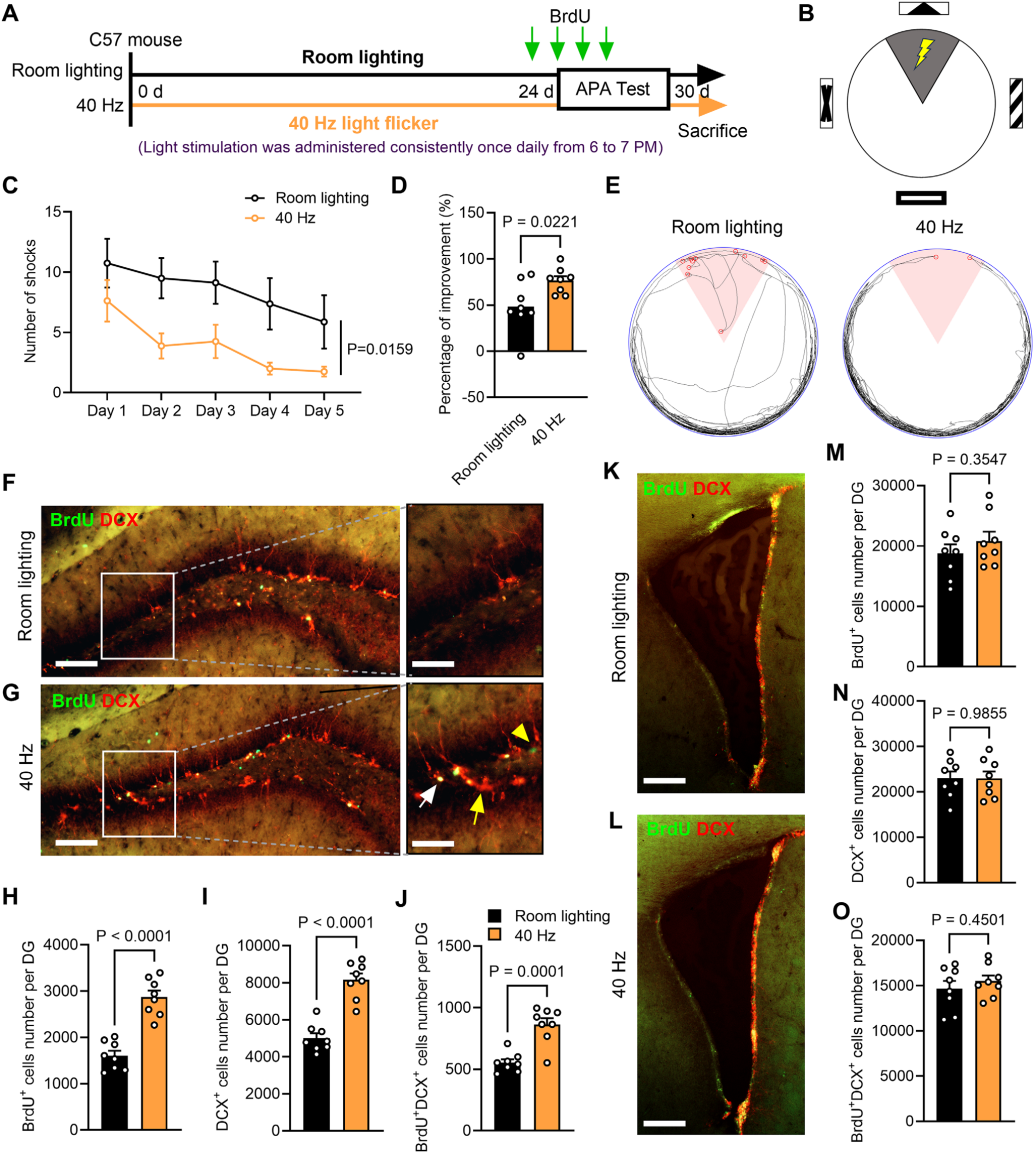
Long‐term 40 Hz light flicker enhances spatial learning and neurogenesis. A) Experimental design and timeline for the Room lighting and 40 Hz groups. B) Schematic diagram of the APA test. C) Quantification of foot shocks received by mice from Room lighting and 40 Hz groups during the APA test [two‐way repeated measures (RM) ANOVA with Tukey's *post hoc* test, F_(1, 14)_ = 7.525, P = 0.0159], showing the effect of long‐term flicker treatment across the days of APA test. *n* = 8 mice per group. D) Percentage improvement in shock avoidance on the 5th day of the APA test compared to the 1st day [two‐tailed unpaired t test with t_(14)_ = 2.573, P = 0.0221]. E) Representative movement trajectory map on the 5th day of the APA test. The 60° pink area indicates the shock zone, and the red circles mark the locations where electric shocks were delivered. F, G) Double immunostaining for BrdU (green) and DCX (red) in the DG of different groups of mice. Boxes indicate enlarged areas. Yellow arrows point to DCX^+^ immature neurons born before BrdU labeling, white arrows indicate BrdU^+^/DCX^+^ cells, and yellow arrowheads show BrdU^+^ cells that are not yet immature neurons. *n* = 8 mice per group. Scale bars, 100 µm. H–J) Quantification of the number of BrdU^+^ cells (H), DCX^+^ cells (I), and BrdU^+^/DCX^+^ cells (J) in the DG [two‐tailed unpaired t test for H: t_(14)_ = 7.137, P < 0.0001; two‐tailed unpaired t test with Welch's correction for I: t_(12.99)_ = 7.208, P < 0.0001; and two‐tailed unpaired t test for J: t_(14)_ = 5.318, P = 0.0001]. K, L) Double immunostaining for BrdU (green) and DCX (red) in the SVZ. Scale bars, 100 µm. M–O) Quantification of the number of BrdU^+^ cells (M), DCX^+^ cells (N), and BrdU^+^/DCX^+^ cells (O) in the SVZ after long‐term 40 Hz light flicker [two‐tailed unpaired t test for M: t_(14)_ = 0.9572, P = 0.3547; for N: t_(14)_ = 0.01847, P = 0.9855; and for O: t_(14)_ = 0.7769, P = 0.4501].

Spatial learning performance was evaluated using the active place avoidance (APA) test from day 25 to day 30 (**Figure** **1**A). In this test, mice were placed on a rotating platform and assessed on their ability to avoid a stationary shock zone using spatial visual cues (Figure 1B). Mice receiving long‐term 40 Hz light flicker treatment showed significant improvement in avoiding the shock zone compared to untreated mice, with better performance from test days 2 to 5 (Figure 1C–E).

Furthermore, a series of behavioral tests were performed to evaluate potential changes in motor and emotional functions following long‐term 40 Hz light flicker treatment (Figure S1A, Supporting Information). The open‐field test (OFT) showed no significant differences in distance traveled or time spent in the center of the field between the Room lighting and 40 Hz groups (Figure S1C, D, Supporting Information). Similarly, the elevated plus‐maze (EPM) test revealed no changes in anxiety levels between the two groups (Figure S1B, E, and F, Supporting Information). The three‐chamber social preference test also indicated that prolonged 40 Hz light flicker did not affect the mice's social interaction or preference for social novelty (Figure S1G—K, Supporting Information). Overall, these findings suggest that long‐term 40 Hz light flicker treatment did not impact locomotion, social behaviors or induce anxiety‐related behaviors.

### 2.2 Long‐Term 40 Hz Light Flicker Significantly Enhances DG Neurogenesis

Given the established link between enhanced spatial learning and increased hippocampal neurogenesis,^[^ ^26^, ^58^, ^59^ ^]^ we sought to determine whether prolonged exposure to 40 Hz light flicker promotes neurogenesis in the adult mouse brain in vivo. Pilot studies were conducted to identify the optimal time window for BrdU injection, allowing for the observation and quantification of newly divided cells labeled with BrdU in the DG following light stimulation.^[^ ^28^, ^60^, ^61^ ^]^ Empirical data indicated that administering BrdU throughout the 30‐day light treatment period was unnecessary, as most newly generated neurons became post‐mitotic and transitioned into immature neurons, which would be labeled with DCX.^[^ ^61^ ^]^ Consequently, we administered BrdU daily during the final week of the light treatment to label the newborn cells within one week (days 24 to 27). The number of labeled newborn cells in the hippocampal DG and subventricular zone (SVZ) was then quantified using immunostaining for BrdU and the immature neuronal marker DCX (Figure 1A). The BrdU^+^ only cells represent the mitotic cells newly generated within one week, the DCX^+^ only cells represent the post‐mitotic cells within one week, and BrdU^+^/DCX^+^ co‐stained cells represent the post‐mitotic cells newly generated within one week.

Compared to untreated mice, those subjected to long‐term 40 Hz light flicker treatment exhibited a significant increase in the number of BrdU^+^, DCX^+^, and BrdU^+^/DCX^+^ co‐stained cells in the DG region (Figure 1F–J). However, no changes occurred in the SVZ region (Figure 1K–O). These results demonstrated that long‐term exposure to 40 Hz light flicker enhanced neurogenesis, specifically in the DG, which could underlie the observed improvements in spatial learning performance.

To investigate whether prolonged treatment with different flicker frequencies also affects neurogenesis in vivo, mice were exposed to 8 Hz, 40 Hz, and 70 Hz flickers for 30 days. All these frequencies resulted in increased neurogenesis in the DG (Figure S2A—D, Supporting Information). Notably, 40 Hz elicited the most significant enhancement of neurogenesis in the DG, indicating a selective frequency response. Moreover, we also investigated whether a 40 Hz audio stimulus affects neurogenesis. Mice exposed to 30 days of 40 Hz audio stimulus showed improved spatial learning and a significant increase in DG neurogenesis (Figure S2E—J, Supporting Information). These results indicate that the 40 Hz audio stimulus has a similar beneficial impact on cognitive function and neurogenesis as the 40 Hz visual stimulus.

Our previous studies, along with those of others, show that frequencies of 30, 40, and 50 Hz do not affect locomotion or anxiety levels in mice^[^ ^6^, ^7^, ^9^, ^10^ ^]^ and provide neuroprotection to CA1 neurons in a two‐vessel occlusion stroke model.^[^ ^9^ ^]^ In contrast, frequencies above 70 Hz induce anxiety‐like behavior.^[^ ^10^ ^]^ Based on these findings, subsequent experiments were conducted using the 40 Hz light flicker.

### 2.3 Enhancement of Spatial Learning Requires DG Neurogenesis Evoked by Light Flicker

**Figure 2:**
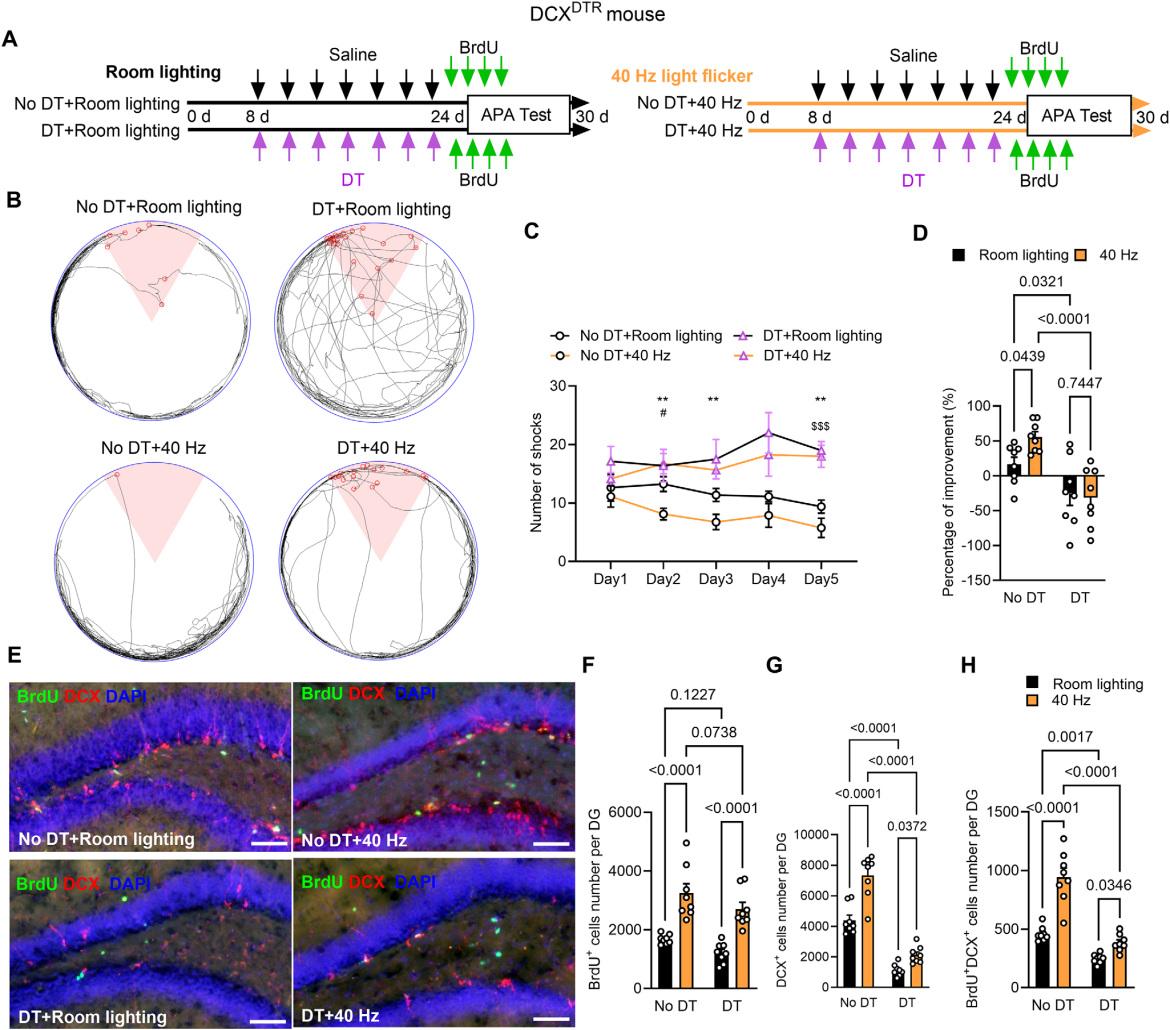
Ablation of DCX^+^ cells prevents the spatial learning enhancement induced by long‐term light flicker. A) Experimental design showing four groups of DCX^DTR^ mice: saline (DCX^DTR^), Diphtheria toxin (DCX^DTR^+DT), 40 Hz light flicker (DCX^DTR^+40 Hz), and 40 Hz light flicker with DT (DCX^DTR^+DT+40 Hz). B) Movement trajectory map on the 5th day of the APA test for the four groups with the 60° pink area for the shock zone and red circles for shock sites. C) Total number of shocks received by the four groups over the 5‐day APA test. D) Percentage improvement in shock avoidance on the 5th day relative to the 1st day of the APA test. Ablation of DCX^+^ cells in adult DCX^DTR^ mice significantly eliminated the spatial learning improvement observed after long‐term 40 Hz light flicker treatment. *n* = 8 mice per group. E) Double immunostaining for BrdU (green) and DCX (red) with DAPI (blue) showing the DG of four groups. Scale bars, 100 µm. F–H) Quantification of BrdU^+^ (F), DCX^+^ (G), and BrdU^+^/DCX^+^ (H) cell numbers in the DG of four groups. *n* = 8 mice for each group. Two‐way ANOVA with Tukey's *post hoc* test was used for panels C, D, and Two‐way ANOVA with Fisher's LSD test for F‐H with P values as indicated on the graph. For panel C, the specific symbols indicate selective comparisons: *P_DCX_^DTR^_+40 Hz versus DCX_^DTR^_+DT+40 Hz_; #P_DCX_^DTR^_versus DCX_^DTR^_+40 Hz_; $P_DCX_^DTR^_versus DCX_^DTR^_+DT_; **P < 0.01, ***P < 0.001; #P < 0.05; $$$P < 0.001.

To investigate whether the enhanced spatial learning from long‐term 40 Hz light flicker treatment depends on adult neurogenesis, we used transgenic knock‐in DCX^DTR^ mice, which express the human diphtheria toxin receptor (DTR) under the control of the DCX promoter.^[^ ^28^ ^]^ Administration of diphtheria toxin (DT) caused deletion of newborn neurons in vivo. Starting on day 8, DCX^DTR^ mice received intraperitoneal (i.p.) injections of either saline or DT every 2 days for 2 weeks (**Figure** **2**A). The OFT showed that neither DT nor 40 Hz light flicker treatment affected anxiety‐like behavior or spontaneous motor activity in the DCX^DTR^ mice (Figure S1L, M, Supporting Information). In addition, to exclude the potential off‐target effects of DT administration, we injected DT into wild‐type mice (C57+DT group) and used saline as a control (C57+Saline group). The number of BrdU+ and DCX+ cells did not show a significant difference between the two groups, indicating that DT did not affect neurogenesis in the hippocampus of wild‐type mice (Figure S3A—C, Supporting Information).

Importantly, the DCX^DTR^+40 Hz group significantly improved their ability to avoid the shock zone during the APA test compared to the DCX^DTR^ group. However, there was no difference in the number of shocks received between the DCX^DTR^+DT+40 Hz group and the DCX^DTR^+DT group, indicating a failure to learn avoidance (Figure 2B–D).

Furthermore, the numbers of BrdU^+^, DCX^+^, and BrdU^+^/DCX^+^ cells were assessed using immunohistochemistry (Figure 2E). The DCX^DTR^+40 Hz group showed a significant increase in the number of BrdU^+^, DCX^+^, and BrdU^+^/DCX^+^ cells in the DG compared to the DCX^DTR^ group (Figure 2E–H). There was no substantial difference in the number of BrdU^+^ cells in the DG between the DCX^DTR^+DT group and the DCX^DTR^ group, suggesting that the deletion of DCX^+^ cells in the adult mouse DG did not affect neural progenitor proliferation (Figure 2F). In contrast, the number of DCX^+^ cells and BrdU^+^/DCX^+^ cells in the DCX^DTR^+DT group was significantly reduced compared to the DCX^DTR^ group, confirming the effectiveness of the DT‐mediated depletion of DCX^+^ cells (Figure 2G, H). Additionally, the DCX^DTR^+DT+40 Hz group did not show a significant change in the number of BrdU^+^ cells, but there was a marked reduction in both DCX^+^ and BrdU^+^/DCX^+^ cells in the DG compared to the DCX^DTR^+40 Hz group (Figure 2F–H).

Collectively, these results demonstrate that the elimination of adult neurogenesis abolishes the spatial learning enhancement induced by 40 Hz light flicker treatment.

### 2.4 Long‐Term 40 Hz Light Flicker Does Not Alter DG Microcirculation

To investigate the mechanisms underlying the long‐term effects of 40 Hz stimulation, we assessed DG regional microvessel blood flow using an implanted GRIN lens and two‐photon microscopy in vivo (Figure S4A, B, Supporting Information). Capillaries were classified into eight branching levels, starting from the initial arteriole (EA). The first level was defined as the capillary immediately following the cessation of the red color hydrazide fluorescent signal (Figure S4C, Supporting Information). Capillaries from the 1st to the 4th level were categorized as “pre‐4th,” and capillaries from the 5th to the 8th level were categorized as “post‐4th” (Figure S4C, Supporting Information).

The results revealed that long‐term exposure to 40 Hz light flicker did not significantly affect the lumen diameter (Figure S4E—G, Supporting Information) or red blood cell velocity (Figure S4H—J, Supporting Information) in either pre‐ or post‐4th level arterioles and capillaries. Additionally, measurements taken during the 40 Hz flicker condition in long‐term treated mice (40 Hz+flickering) indicated that the sensitivity of DG microvessels to light flicker remained unchanged (Figure S4E—J, Supporting Information). These findings suggest that prolonged 40 Hz light flicker stimulation does not induce alterations in regional microcirculation within the DG.

### 2.5 Activation of DG PV Interneurons by 40 Hz Flicker

**Figure 3:**
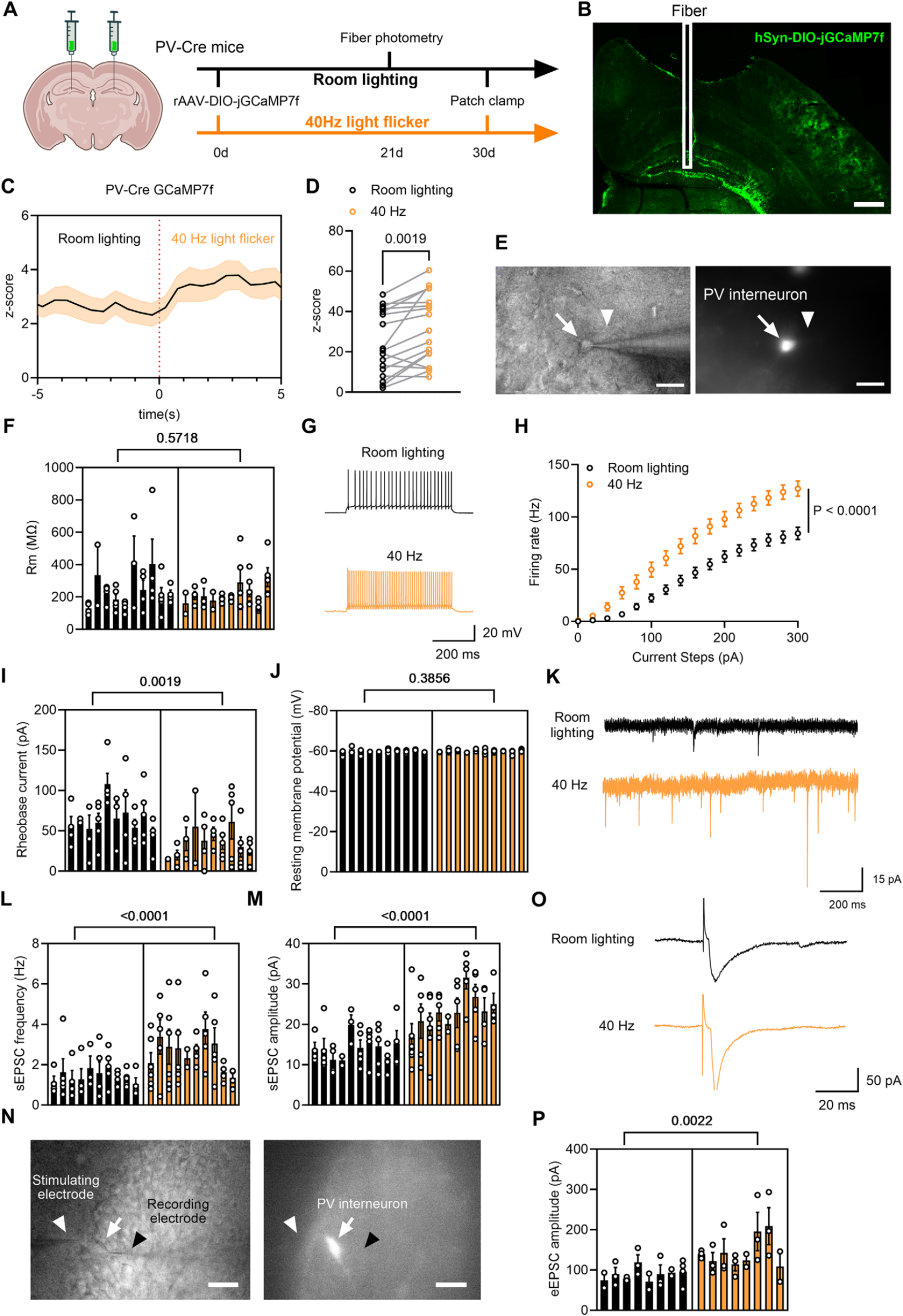
Long‐term flicker activates PV interneurons in the DG. A) Diagram showing viral injection sites in PV^Cre^ mice and experimental timeline for fiber photometry and patch clamp. B) Image of brain coronal section showing the recording probe track and fluorescent rAAV expression in DG. Scale bar, 200 µm. C) Fiber photometry of PV interneuron GCaMP7f signals in response to 40 Hz light flicker. Average z‐score of signals 5 seconds before and during flicker. *n* = 6 mice. D) Quantification of PV interneuron GCaMP7f intensity before and during 40 Hz flicker [two‐tailed paired t‐test with t(16) = 3.700, P = 0.0019]. E) Images of fluorescent labeled PV interneurons near the DG granule cell layer for patch clamp recording. Arrowhead: glass pipette; Arrows: PV interneurons. Scale bar, 20 µm. F) Quantification of PV interneuron membrane resistance (Rm) [nested two‐tailed unpaired t test, t(76) = 0.5759, P = 0.5718]. G) Representative traces for firing rate of PV interneurons. H) Quantification of firing rate of PV interneurons after long‐term 40 Hz flicker stimulation [two‐way RM ANOVA with Tukey's post hoc test with F(1, 76) = 19.87, P < 0.0001], showing a significant interaction between the effects of 40 Hz stimulation and firing rate. I) Significant decrease in rheobase current after prolonged 40 Hz flicker [nested two‐tailed unpaired t test with t(76) = 3.640, P = 0.0019]. J) Quantification of resting potential of PV interneurons after long‐term 40 Hz flicker stimulation [nested t two‐tailed unpaired t test with t(76) = 0.8892, P = 0.3856]. K) Representative sEPSCs traces recorded in PV interneurons. L, M) Quantifications of sEPSC frequency (L) and amplitude (M) [nested two‐tailed unpaired t test for l: t(89) = 4.570, P < 0.0001; and for M: t(18) = 5.594, P < 0.0001]. N) Images of patch clamp recording for fluorescent PV interneurons near the DG granule cell layer, showing the recording pipette (black arrowheads), stimulating pipette (white arrowheads), and recorded PV interneurons (white arrows). Scale bar, 20 µm. O) Representative traces of eEPSCs. P) Quantification of eEPSC amplitude [nested two‐tailed unpaired t test with t(14) = 3.744, P = 0.0022]. For panels F, H, I, *n* = 10 mice, 40 cells recorded for the Room lighting group; *n* = 10 mice, 40 cells recorded for the 40 Hz group. For panels L, M, *n* = 10 mice, 44 cells recorded for the Room lighting group; *n* = 10 mice, 47 cells recorded for the 40 Hz group. For panels p, *n* = 8 mice, 21 cells recorded for the Room lighting group; *n* = 8 mice, 22 cells recorded for the 40 Hz group.

Fiber photometry was used to investigate whether 40 Hz flicker activates PV interneurons in the DG. Transgenic PV^Cre^ mice received stereotactic injections of rAAV2/9‐hSyn‐DIO‐jGCaMP7f‐WPRE‐pA (referred to as hSyn‐DIO‐jGCaMP7f in **Figure** **3**A) in the DG region in vivo. The GCaMP7f fluorescence signals from PV interneurons were captured using fiber photometry (Figure 3A, B). The analysis showed a significant increase in GCaMP7f fluorescence intensity following the 40 Hz flicker, indicating activation of PV interneurons (Figure 3C,D).

To further investigate the effects of prolonged 40 Hz light flicker exposure on the electrophysiological properties of PV interneurons, we conducted a series of experiments using transgenic PV^Cre^ mice. These mice received stereotactic injections of rAAV‐hSyn‐DIO‐jGCaMP7f into the DG region in vivo, followed by 30 days of 40 Hz light flicker exposure. Afterward, brain slices were prepared for patch‐clamp recordings of PV interneurons in vitro (Figure 3E). Interestingly, long‐term exposure to 40 Hz light flicker did not alter the membrane resistance and resting potential of PV interneurons (Figure 3F,J), there was an increase in firing rates (Figure 3G,H) and a marked reduction in the rheobase currents (Figure 3I) in green fluorescent protein (GFP)‐labeled PV interneurons, indicating that long‐term 40 Hz light flicker treatment lowered the activation threshold and enhanced the excitability of PV interneurons.

To assess the impact on synaptic input to PV interneurons, we measured both spontaneous (sEPSC) and evoked (eEPSC) excitatory postsynaptic currents in PV interneurons after prolonged 40 Hz flicker exposure. Whole‐cell recordings, conducted in the presence of 20 µM bicuculline in artificial cerebrospinal fluid (aCSF) to block inhibitory currents, revealed a significant increase in both sEPSCs (Figure 3K–M) and eEPSCs (Figure 3N–P) in mice exposed to long‐term 40 Hz flicker. These results demonstrated that prolonged 40 Hz flicker stimulation enhances the excitatory input to PV interneurons, further contributing to their increased excitability.

Together, these findings indicate that long‐term exposure to 40 Hz light flicker enhances the intrinsic excitability of PV interneurons and strengthens their excitatory synaptic input, likely contributing to their increased activity in response to the stimulation.

### 2.6 Determining the Role of PV Interneurons in Driving DG Neurogenesis in Response to Light Flicker

**Figure 4:**
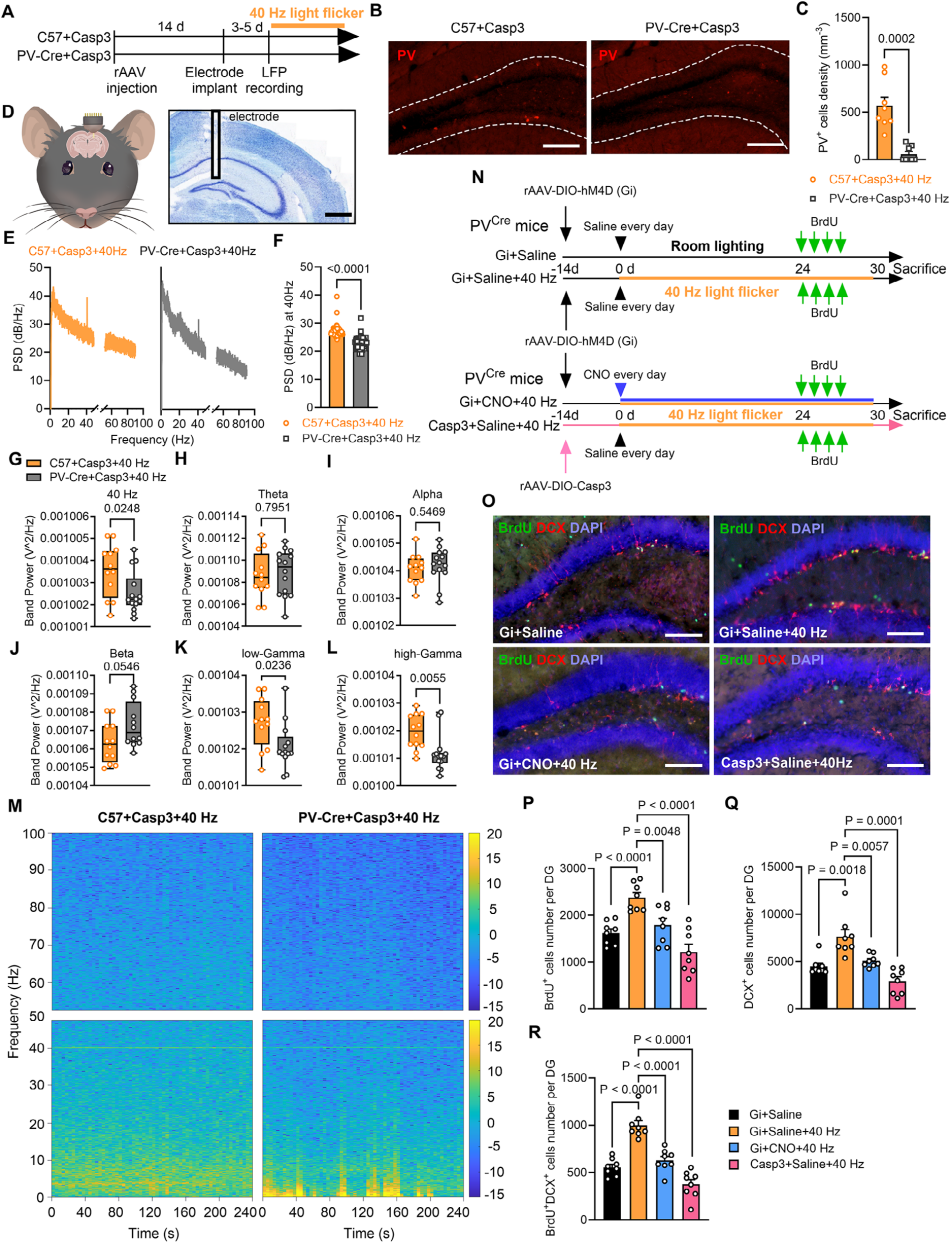
Inactivation of PV interneuron reduces 40 Hz entrainment and neurogenesis in DG. A) Experimental schema for PV deletion groups and LFP. B) Representative PV immunostaining in DG from the control mice (C57+Casp3+40 Hz) and PV deletion mice (PV‐Cre+Casp3+40 Hz). Scale bar, 100 µm. C) Significant reduction in PV interneuron density in PV‐Cre+Casp3 mice compared to C57+Casp3 control mice after long‐term 40 Hz flicker [two‐tailed unpaired t‐test with Mann‐Whitney test, P = 0.002]. *n* = 15 brain slices from 5 mice per group. D) A diagram of electrode insertion in the hippocampus. E) Power spectral density (PSD) of LFP recorded from the DG molecular layer of C57+Casp3+40 Hz and PV‐Cre+Casp3+40 Hz mice during 40 Hz light flicker. F) Quantification of PSD during 40 Hz light flicker [two‐tailed unpaired t‐test with Mann‐Whitney test, P < 0.0001]. G–L) Band power analysis showing significant reduced 40 Hz (G), low gamma (K), and high gamma (L) power in PV‐Cre+Casp3+40 Hz mice compared to C57+Casp3+40 Hz mice during 40 Hz light flicker. No significant differences occurred in theta (H), alpha (I), or beta (J) band power between the two groups. A two‐tailed unpaired t test was performed for G [t_(24)_ = 2.394, P = 0.0248], H [t_(24)_ = 0.2626, P = 0.7951], I [t_(24)_ = 0.6110, P = 0.5469], J [t_(24)_ = 2.031, P = 0.0546], K [t_(24)_ = 2.425, P = 0.0236], and L [t_(24)_ = 3.052, P = 0.0055]. M) STFT heatmap of LFP recorded in DG over 240 seconds during resting states, with PSD intensity color‐coded as per the scale bars on the right. N) Experimental schema for chemogenetic inactivation of PV interneurons using inhibitory virus AAV‐DIO‐hM4D(Gi) and deletion of PV interneurons using caspase‐3 (AAV‐DIO‐Casp3) in PV^Cre^ mice. O) Double immunostaining for BrdU (green), DCX (red), and DAPI (blue) in the DG of four mouse groups. Scale bar, 100 µm. P–R) Chemogenetic inactivation and caspase‐3 deletion of PV interneurons significantly decreased the number of BrdU^+^ cells (P), DCX^+^ cells (Q), and BrdU^+^/DCX^+^ cells (R) in the DG. Student's t test was performed for panels P‐R, with specific P values as indicated. *n* = 8 mice per group.

Two approaches were used to determine the necessity of PV interneurons in promoting neurogenesis in the DG following prolonged 40 Hz stimulation. First, PV interneurons were selectively deleted in PV^Cre^ mice by injecting them with the rAAV2/9‐CAG‐DIO‐taCaspase3‐TEVp‐WPRE‐pA virus (rAAV‐DIO‐Casp3), which expresses caspase‐3 (referred to as PV‐Cre+Casp3 in **Figure** **4**). In contrast, the PV^Cre^ wild‐type littermates, which lack Cre recombinase expression, received the same rAAV‐Caspase3‐pA virus and served as the caspase‐3 negative control (C57+Casp3) (Figure 4A). Immunohistochemical staining of the mouse hippocampus revealed a significant reduction in the number of PV interneurons in the DG region of the PV‐Cre+Casp3 group (Figure 4B,C).

Local field potentials (LFPs) were recorded in mouse DG molecular layer with PV interneurons selectively deleted in vivo (Figure 4D; Figure S5A, B, Supporting Information). In the C57+Casp3 control group, exposure to 40 Hz light flicker resulted in an increase in power spectral density (PSD) at 40 Hz (Figure 4E–G) and enhanced LFP oscillations, as shown in the time‐resolved spectrogram (Figure 4M). In contrast, the PV‐Cre+Casp3 group exhibited a significant reduction in 40 Hz PSD (Figure 4E–G) and in gamma frequency oscillations (30‐100 Hz) (Figure 4K and L), while theta (4–8 Hz), alpha (8–12 Hz), and beta (13–30 Hz) oscillations remained unaffected (Figure 4H–j). These findings underscore the crucial role of PV interneurons in mediating 40 Hz entrainment and gamma oscillations within the DG.

Second, a chemogenetic approach was used to inactivate PV interneurons in vivo. PV^Cre^ mice received stereotactic injections of rAAV2/9‐hSyn‐DIO‐hM4D(Gi)‐eGFP‐WPRE‐pA (hSyn‐DIO‐Gi) virus (referred to as Gi+CNO+40 Hz groups in Figure 4N). Clozapine N‐oxide (CNO) was administered continuously throughout the flicker treatment with saline served as a control (Gi+Saline and Gi+Saline+40 Hz groups) (Figure 4N). Over 80% of virus‐infected neurons are PV interneurons (Figure S5C, D, Supporting Information). The chemogenetic inactivation of PV interneurons in the Gi+CNO+40 Hz group significantly inhibited PV firing rate after long‐term 40 Hz flicker treatment, as demonstrated by patch‐clamp recordings (Figure S5E—J, Supporting Information).

Importantly, both approaches substantially reduced the neurogenesis induced by long‐term 40 Hz light flicker treatment, evidenced by decreased numbers of BrdU^+^, DCX^+^, and BrdU^+^/DCX^+^ cells in groups of Gi+CNO+40 Hz and Casp3+Saline+40 Hz (which was the same as PV+Casp3 group as described above) (Figure 4O–R). These findings suggest that deletion or inactivation of PV interneurons impairs the neurogenesis promoted by 40 Hz stimulation in the DG.

Despite prolonged exposure to 40 Hz light flicker, the numbers of PV interneurons (Figure S6A, B, Supporting Information) and Tbr2^+^ cells (Figure S6C, D, Supporting Information) remained unchanged, indicating that 40 Hz light flicker stimulation did not directly affect the proliferation of Tbr2^+^ intermediate neural progenitor cells in the adult DG. Conversely, the deletion and inactivation of PV interneurons significantly decreased the number of Tbr2^+^ cells in the DG (Figure S6E, Supporting Information). Collectively, these results suggest that neurogenesis in the adult DG induced by 40 Hz light flicker depends on the functional presence of PV interneurons, which likely support immature neurons in the DG rather than directly influencing the proliferation of neural progenitors.

Finally, to further demonstrate that activation of PV interneurons alone is sufficient to promote neurogenesis, we injected the rAAV2/9‐hSyn‐DIO‐hM3D(Gq)‐eGFP‐WPRE‐pA (rAAV‐DIO‐hM3D) virus into transgenic PV^Cre^ mice (Figure S7, Supporting Information). CNO was administered continuously for 30 days (Gq+CNO), with saline serving as a control (Gq+Saline) (Figure S7A, Supporting Information). Over 85% of the virus‐infected neurons were PV interneurons (Figure S7D, E, Supporting Information). We found that PV activation enhanced both neurogenesis and spatial learning abilities in the Gq + CNO mice (Figure S7B, C, and F—I, Supporting Information).

### 2.7 Induction of GABA Levels in the DG by 40 Hz Flicker Stimulation

It has been extensively studied that activation of PV interneurons promoted the survival and development of newborn neurons through enhanced release of inhibitory neurotransmitter GABA.^[^ ^39^, ^45^, ^61^, ^62^ ^]^ We, therefore, examined changes in GABA levels within the DG to understand better how PV interneurons support neurogenesis during light flicker treatment.

**Figure 5:**
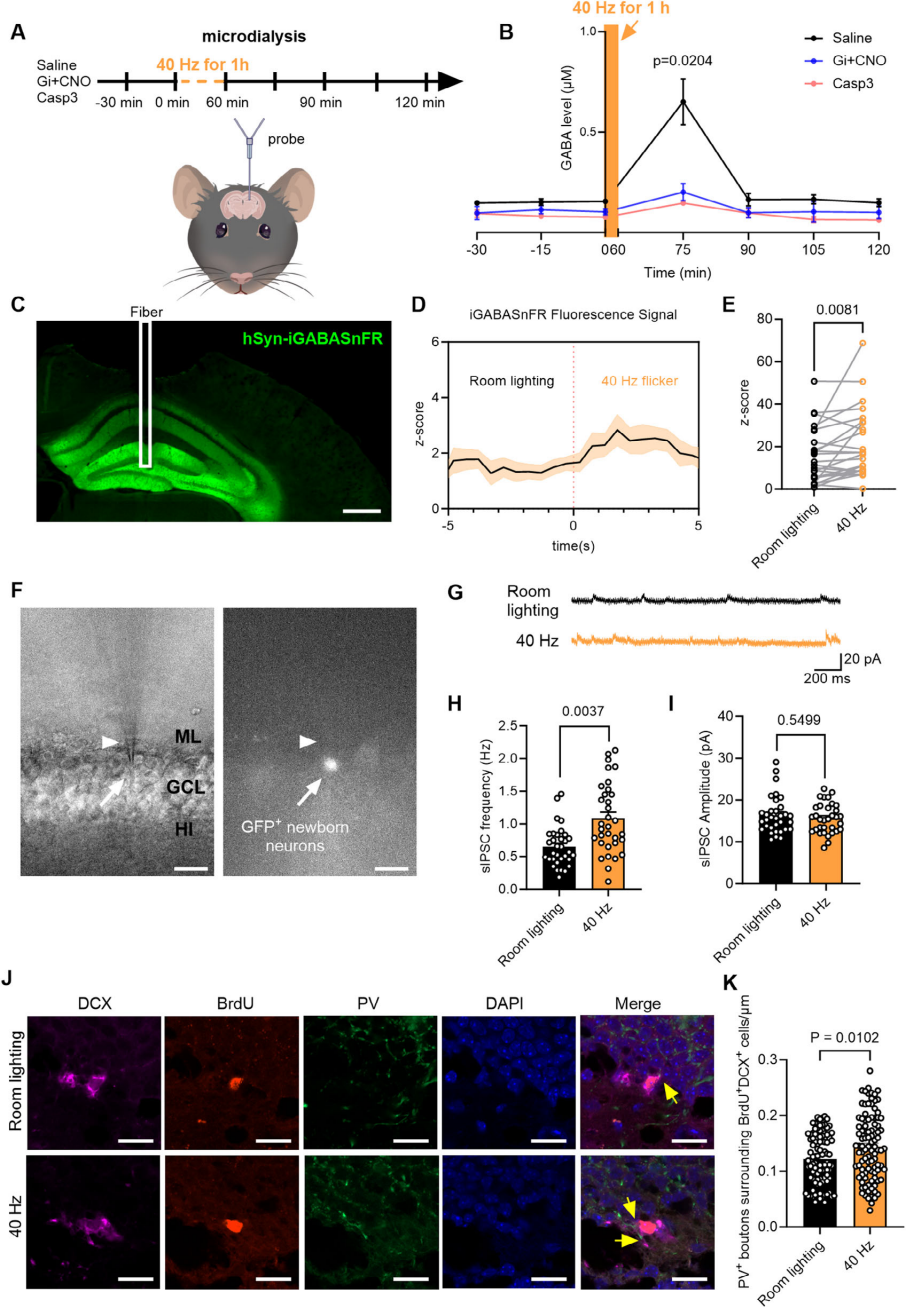
Increased GABA level in DG evoked by 40 Hz light flicker. A) Experimental timeline for collecting microdialysates from the DG. B) HPLC analysis and quantification of microdialysates during and after 1 hour of 40 Hz light flicker [one‐way ANOVA with F_(2, 7)_ = 7.142, P = 0.0204]. C) Image of brain coronal section showing the recording probe track and fluorescent GABA sensor. Scale bar, 200 µm. D) Fiber photometry of iGABAFnSR fluorescence signals was plotted as the average z‐score for 5 seconds before and 5 seconds during the flicker. n = 7 mice. E) Quantification of iGABAFnSR fluorescence intensity [two‐tailed paired t‐test with Wilcoxon matched‐pairs signed rank test, P = 0.0081]. F) Electrophysiological recordings of DCX^+^ neurons expressing eGFP using rVSVG‐Retrovirus. DCX^+^ neurons were identified via phase contrast (left) and epifluorescent imaging (right). Scale bar, 20 µm. ML = molecular layer, GCL = granule cell layer, HI = hilus. G) Representative traces of firing rate recorded in DCX^+^ neurons. H, I) Quantification of DCX^+^ neuron sIPSC frequency (H) and amplitude (I) [nested two‐tailed unpaired t test for H: t(14) = 3.474, P = 0.0037; and for I: t(14) = 0.6127, P = 0.5499]. J) Confocal images showing PV^+^ (green) perisomatic puncta (yellow arrows) on BrdU^+^ (red) and DCX^+^ (violet) double‐labeled cells in different groups. Scale bar, 20 µm. K) Quantification of PV^+^ puncta density in the perisomatic region of BrdU^+^/DCX^+^ cells after 40 Hz light flicker [two‐tailed unpaired t‐test with Mann‐Whitney test, P = 0.0102]. *n* = 98 cells from 3 mice per group. For panels H and I, *n* = 22 cells recorded from 5 mice for the Room lighting group and the 40 Hz group, respectively.

Microdialysis was employed to collect microdialysate within the DG region. To monitor alterations in GABA levels in vivo, which was subsequently analyzed for GABA concentrations using high‐performance liquid chromatography (HPLC). Samples were collected every 15 minutes, starting 45 minutes before the 1‐hour 40 Hz light flicker stimulation session and continuing for 60 minutes after the treatment (**Figure** **5**A,B).

Following the 1‐hour 40 Hz light flicker treatment, the GABA levels in the DG of the Saline group showed a significant increase, with levels returning to baseline within 30 minutes after the conclusion of the flicker treatment (Figure 5B). To determine whether these changes in GABA levels were dependent on PV activity, PV^Cre^ mice were injected rAAV‐DIO‐Gi followed by CNO treatment (referred to as Gi+CNO in Figure 5B) or rAAV‐DIO‐Casp3 (referred to as Casp3 group in Figure 5B). These groups of mice showed no significant change in GABA levels following the 40 Hz stimulation.

These results demonstrate that prolonged 40 Hz light flicker stimulation increases GABA levels within the DG region, an effect that is abolished when PV interneurons are either inactivated (via Gi+CNO) or deleted (via Caspase‐3). Therefore, PV interneurons play a crucial role in modulating GABAergic responses to 40 Hz stimulation within the DG.

To evaluate the temporal changes in GABA levels during 40 Hz flicker, we performed an immunohistochemical analysis to measure GABA expression in the DG at several time points: Room lighting, 15 minutes of flicker, 30 minutes of flicker, and 30 minutes after the 1‐hour flicker session (Figure S8A, Supporting Information). The results revealed a significant increase in GABA intensity within the DG during the first 30 minutes of the 40 Hz flicker. GABA levels of the Room lighting group returned to baseline 30 minutes following the conclusion of the 1‐hour flicker treatment (Figure S8B, Supporting Information).

To unequivocally demonstrate increased GABA levels, we used a specific GABA adenoviral sensor, rAAV2/9‐hSyn‐iGABASnFR‐WPRE‐pA (referred to as hSyn‐iGABASnFR in Figure 5), in combination with fiber photometry to measure real‐time changes in GABA levels within the DG. The GABA sensor was injected into the hilus of the DG and allowed to express for 3 weeks (Figure 5C). The resulting in vivo fluorescent signal from the GABA sensor was processed, and the robust z‐scores revealed a significant increase in GABA levels during 40 Hz flicker stimulation (Figure 5D,E). Together, these findings demonstrate that 40 Hz flicker stimulation effectively elevates GABA levels in the DG.

### 2.8 Promotion of Enhanced Network Integration of Newborn DCX+ Cells by 40 Hz Flicker

To understand whether flicker promotes the integration of newborn DCX^+^ cells into hippocampal circuits, we examined enhanced presynaptic inhibition received from GABAergic neurons onto newborn neurons. Mice were injected with rVSVG‐Retrovirus‐CAG936‐eGFP‐3xFlag‐WRPE‐pA (rVSVG‐Retrovirus) in the DG to label newborn DCX^+^ neurons with GFP (Figure 5F). Retroviral vectors expressing GFP are effective pulse markers for dividing cells, making them helpful for tracking the fate of progeny from a specific cell after a mitotic event.^[^ ^63^ ^]^ Using patch clamp recording in vitro, we observed a significant increase in the frequency of sIPSCs in these GFP^+^ cells, while the sIPSC amplitude remained unchanged following the flicker (Figure 5G–I). This data indicates that the newborn neurons received enhanced presynaptic inhibitory input from GABAergic interneurons after prolonged 40 Hz flicker.

Furthermore, we assessed the density of perisomatic synaptic puncta formed by PV interneurons surrounding BrdU^+^/DCX^+^ cells (Figure 5J) to demonstrate whether 40 Hz light flicker treatment enhances PV GABAergic synaptic input onto newborn neurons. Indeed, a significant increase in the density of PV^+^ perisomatic synaptic puncta around BrdU^+^/DCX^+^ cells in mice exposed to prolonged 40 Hz light stimulation occurred (Figure 5K). Combined with microdialysis data, these findings demonstrate that prolonged 40 Hz light stimulation enhanced the GABA level and promoted better integration of newborn DCX^+^ cells.

Interestingly, prolonged 40 Hz flicker did not promote the maturation process of newborn DCX^+^ cells. The membrane resistance, resting membrane potential, rheobase current, and firing rate of newborn DCX^+^ cells labeled with retroviral vectors expressing GFP, remained unchanged after the 40 Hz flicker (Figure S9A—G, Supporting Information). Additionally, there were also no significant changes in the frequency or amplitude of sEPSCs (Figure S9H—J, Supporting Information), indicating that 40 Hz flicker does not alter the maturation process of newborn DCX^+^ cells.

### 2.9 Blocking GABAAR Abolishes Enhancement of Neurogenesis and Spatial Learning

**Figure 6:**
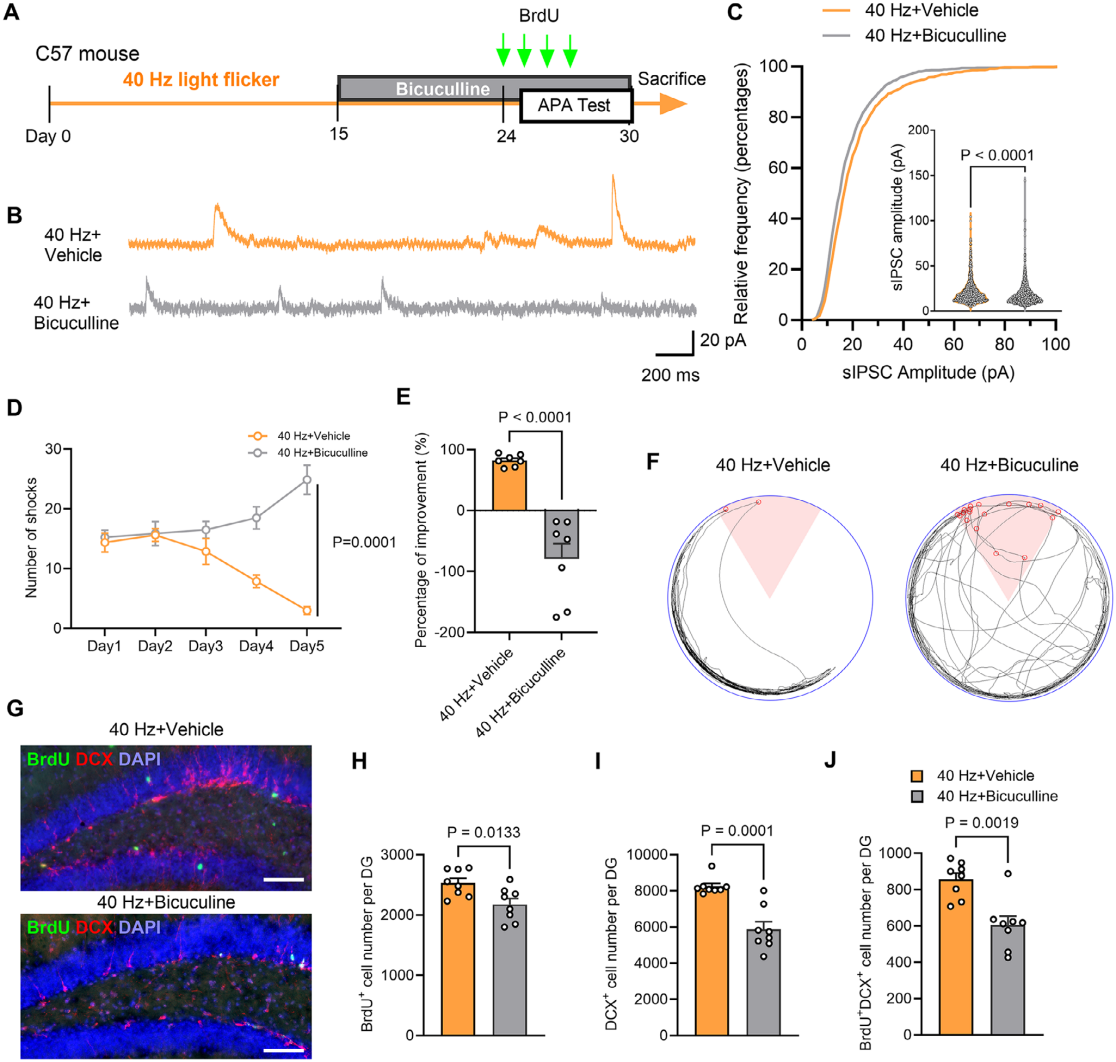
Inhibition of GABA_A_R in adult mice abolishes enhanced spatial learning. A) Experimental schema for GABA_A_R antagonist treatment and APA test. B) Representative traces of firing rate from DCX^+^ neurons. C) Bicuculline treatment significantly reduced the amplitude of sIPSCs in granule cells after 40 Hz light flicker exposure [two‐tailed Mann‐Whitney test with P < 0.0001]. D) Mice treated with bicuculline received significantly more shocks during the 5‐day APA test. Two‐way RM ANOVA with Tukey's *post hoc* test revealed a significant effect of 40 Hz light flicker and bicuculline treatment on shock frequency [F_(12, 1)_ = 30.74, P = 0.0001]. *n* = 7 mice per group. E) Percentage improvement in avoiding shocks on the 5th day of the APA test compared to the 1st day [two‐tailed unpaired t‐test with t_(12)_ = 6.306, P < 0.0001]. F) Representative movement trajectory map from the 5th day of the APA test. G) Double immunostaining of BrdU (green) and DCX (red) with DAPI (blue) of the DG region. Scale bars, 100 µm. H–J) Bicuculline treatment significantly decreased the number of BrdU^+^ cells (H), DCX^+^ cells (I), and BrdU^+^/DCX^+^ cells (J) in the DG after 40 Hz light flicker [two‐tailed unpaired t test for H: t_(14)_ = 2.832, P = 0.0133; two‐tailed unpaired t‐test with Mann‐Whitney test for I: t_(14)_ = 5.222, P = 0.0003; and two‐tailed unpaired t test for J: t_(14)_ = 4.174, P = 0.0009]. *n* = 8 mice per group.

To determine the role of 40 Hz light flicker evoked GABA, we blocked GABA_A_Rs using bicuculline in vivo. Mice received daily bicuculline injections for 15 days (**Figure** **6**A). The amplitude of sIPSCs in granule cells from the 40 Hz+Bicuculline group was significantly reduced compared to the 40 Hz+Vehicle group recorded using brain slices in vitro (Figure 6B, C). In the APA test in vivo (days 25–30), mice in the 40 Hz+Vehicle group showed fewer shocks on the last two testing days, while the 40 Hz+Bicuculline group failed to learn the shock zone location by day 5 (Figure 6D–F). Additionally, the 40 Hz+Bicuculline group had significantly fewer BrdU^+^, DCX^+^, and BrdU^+^/DCX^+^ cells in the DG compared to the 40 Hz+Vehicle group (Figure 6G–J). These results suggest that blocking GABA_A_Rs abolished the neurogenesis and spatial learning enhancements induced by long‐term 40 Hz light stimulation.″

### 2.10 Flicker Stimulation Does Not Affect Cholinergic Activity in the DG or the Activity of VIP and CCK Interneurons

Previous work from Dr. György Buzsák group showed that 40 Hz flicker stimulation induces avoidance behavior and slightly elevates cholinergic activity in the hippocampus at about 0.5% (*ΔF/F*%).^[^ ^14^ ^]^ Given the link between cholinergic modulation and neurogenesis, we investigated whether long‐term 40 Hz flicker stimulates cholinergic activity. To do this, we injected a sensitive cholinergic adenoviral sensor rAAV2/9‐hSyn‐GRABeen‐ACh3.0‐WPRE‐pA (referred to as hSyn‐GRABeen Ach3.0 in Figure S6C, Supporting Information) into the DG and used fiber photometry to measure changes in acetylcholine levels in vivo (Figure S6D, Supporting Information). The results showed no significant increase in cholinergic activity during 40 Hz flicker stimulation (Figure S6E, Supporting Information). Additionally, long‐term flicker treatment did not induce anxiety or emotional behavior defects in mice (Figure S1, Supporting Information), suggesting that cholinergic‐driven neurogenesis is unlikely a major contributor to neurogenesis during long‐term flicker stimulation.

Given the complexity of hippocampal circuitry, it is important to investigate whether other DG interneurons, such as VIP and CCK, are also affected by 40 Hz light flicker. To address this, we used VIP‐Cre and CCK‐Cre mice injected with rAAV‐DIO‐jGCaMP7f virus, respectively (referred to as hSyn‐DIO‐jGCaMP7f in Figures S10A and S11A, Supporting Information) into the DG to label respective interneurons. We then measured the calcium activity and neuronal excitability of VIP and CCK interneurons before and after exposure to 40 Hz light flicker to determine whether these interneurons were activated in vivo. Fiber photometry in the DG was performed (Figures S10B and S11B, Supporting Information). The results showed no significant increase in calcium activity in VIP and CCK interneurons during 40 Hz flicker stimulation (Figures S10C and S11C, Supporting Information).

To further confirm these findings, we conducted patch clamp recordings on VIP and CCK interneurons in brain slices in vitro (Figures S10D, S11D, Supporting Information). These recordings showed that prolonged 40 Hz flicker did not alter the activity of VIP and CCK interneurons. Specifically, membrane resistance, resting membrane potential, rheobase current, and firing rate of these interneurons were unchanged following 40 Hz flicker (Figure S10E–I and S11E—I, Supporting Information). Additionally, there were no significant changes in the frequency or amplitude of sEPSCs in these interneurons (Figure S10J–L and S11J—L, Supporting Information). Together, these data indicate that 40 Hz light flicker does not affect the neuronal excitability of CCK and VIP interneurons.

## 3 Discussion

The present study investigated the effects and mechanisms of long‐term exposure to 40 Hz light flicker. Our results indicate that this prolonged treatment enhances the excitability of PV interneurons and increases GABA levels in the DG, thereby creating a supportive environment for neurogenesis and improving spatial learning. Long‐term exposure to flicker did not affect sociability or elevate stress and anxiety levels in mouse behavioral tests, suggesting its translational potential for human studies.

Neurogenesis in the DG is essential for the enhancement of spatial learning following long‐term exposure to 40 Hz light flicker. This is demonstrated by the reversal of spatial learning improvements when newborn DCX^+^ immature neurons in the DG are eliminated using adult DCX^DTR^ transgenic mice. Notably, 40 Hz light flicker did not affect neurogenesis in the SVZ, further highlighting the selectivity of the 40 Hz light flicker stimulation. Given that adult neurogenesis occurs within a complex local niche that supports neural precursor cells, the SVZ may not respond to 40 Hz light flicker due to the distinct characteristics of its microenvironment. We also found that both 8 Hz and 70 Hz light flicker promoted neurogenesis, although to a lesser extent than 40 Hz flicker. Additionally, 40 Hz audio stimulation increased neurogenesis in the DG. However, it remains unclear whether combining 40 Hz audio and visual stimulation would produce a synergistic effect in neurogenesis.

The optimal time window for BrdU injection, enabling the observation and quantification of newly divided cells labeled with BrdU in the DG following light stimulation, was determined empirically and guided by prior literature.^[^ ^64^ ^]^ Specifically, Cameron and McKay reported that young adult rats exhibit approximately 9400 dividing cells, with a cell cycle duration of 25 hours, leading to the generation of around 9000 new cells per day. Furthermore, within 5 to 12 days following BrdU injection, 50% of all BrdU‐labeled cells in the DG could be identified using neuron‐specific antibodies.^[^ ^64^ ^]^ Based on these findings, our BrdU protocol was designed to facilitate the quantifiable assessment of neurogenesis induced by light stimulation within this time window and in alignment with those published studies.^[^ ^60^, ^61^ ^]^

Prolonged 40 Hz light flicker treatment activates PV interneurons in the DG. Unexpectedly, long‐term exposure to 40 Hz light flicker did not increase the number of PV interneurons, but it enhanced their excitability. Fiber photometry data revealed significantly increased calcium signals in PV interneurons after 40 Hz flicker (Figure 3). In vitro electrophysiology results showed decreased rheobase currents, increased firing rates, and elevated sEPSCs and eEPSCs in PV interneurons following prolonged flicker treatment, indicating enhanced excitability. Furthermore, the increased eEPSCs and sEPSCs reflected enhanced strength and dynamics of excitatory synaptic transmission within the PV interneuron networks.

Eliminating PV interneurons through caspase‐3 expression significantly dampened gamma oscillations in the DG, as gamma rhythmic flicker primarily synchronizes with fast‐spiking PV interneurons. Inhibiting PV interneurons using an rAAV‐DIO‐Gi inhibitory virus markedly abolished the increased level of neurogenesis evoked by prolonged exposure to 40 Hz light flicker. These findings unequivocally demonstrate that PV interneurons are activated during extended 40 Hz light flicker treatment, and their activity is essential for both neurogenesis and the enhancement of cognitive function.

Given the complexity of hippocampal circuitry, it's important to investigate whether 40 Hz light flicker affects other DG interneurons. We provided evidence to show that the calcium activity and electrophysical properties of VIP and CCK interneurons did not change indicating that these two types of interneurons are not affected by 40 Hz light flicker. Indeed, recent studies have shown that light flicker predominantly entrains fast‐spiking interneurons.^[^ ^65^ ^]^ Optotagging experiments identify these neurons as either PV interneurons or narrow‐waveform SST interneurons.^[^ ^65^, ^66^ ^]^ Additionally, studies of mossy cells have shown that their support for DCX^+^ cells is secondary to that provided by PV interneurons, as newborn neurons receive glutamatergic synaptic inputs approximately one week later than GABAergic inputs.^[^ ^66^ ^]^ Nevertheless, newborn neurons must receive NMDA receptor‐dependent input to be successfully integrated into the circuit; otherwise, they will be pruned from the network.^[^ ^30^ ^]^ Therefore, the electrophysiological properties of mossy cells following prolonged 40 Hz light flicker require further investigation.

Does prolonged 40 Hz light flicker enhance the integration of newborn DCX^+^ cells into hippocampal circuits? Our assessment of sIPSCs in GFP‐expressing cells, induced by light flicker following rVSVG‐Retrovirus retrograde labeling, revealed an increase in presynaptic inhibitory input from GABAergic interneurons to the newborn neurons. Additionally, we observed an increase in the density of perisomatic synaptic puncta formed by PV interneurons around BrdU^+^/DCX^+^ cells (Figure 5J), further supporting the hypothesis that 40 Hz light flicker enhances the circuit integration of new neurons. In contrast, no changes were observed in the membrane resistance, resting membrane potential, rheobase current, or firing rate of retroviral GFP‐labeled newborn DCX+ cells after exposure to 40 Hz flicker. This data suggests that light flicker does not promote their maturation. It is plausible to hypothesize that the enhanced integration of newborn neurons contributes to their survival, thereby promoting neurogenesis, though this requires further investigation.

Previous studies using 1 hour of 40 Hz light stimulation (with 1 hour of continuous light stimulation as a sham control) found that mice tended to avoid the 40 Hz flicker, which correlated with a 0.5% (*ΔF/F*%) increase in cholinergic activity in the hippocampus, as determined using GRABeen Ach3.0 fiber photometry.^[^ ^14^ ^]^ Interestingly, the sham control of 1 hour of continuous light stimulation also elicited a transient 0.5% (*ΔF/F*%) elevation in cholinergic activity. It remains unclear whether this level of increase in cholinergic activity is sufficient to promote neurogenesis in the DG. Given that enhanced cholinergic activity is known to promote DG neurogenesis,^[^ ^67^ ^]^ we also conducted fiber photometry measurements of cholinergic activity using GRABeen Ach3.0.^[^ ^68^ ^]^ However, our data showed that exposure to 40 Hz flicker did not alter cholinergic receptor activity. Furthermore, long‐term flicker treatment did not induce anxiety or emotional behavior defects in mice, suggesting that cholinergic network activity in the DG is unlikely to be a major factor in promoting neurogenesis.

In this study, we demonstrated that PV interneurons are both necessary and sufficient to promote neurogenesis in response to light flicker. What factors could potentially be driving these results? We hypothesize that 40 Hz stimulation may enhance the release of brain‐derived neurotrophic factor (BDNF), which, in turn, could promote neurogenesis by activating its receptor, TrkB. This hypothesis is supported by both our preliminary data and existing literature, which show that 40 Hz stimulation increases BDNF levels in models of posttraumatic stress disorder, Alzheimer's disease, and in wild‐type mice.^[^ ^69^, ^70^ ^]^

Taken together, this study demonstrates that long‐term exposure to 40 Hz light flicker activates PV interneurons in the DG, promoting neurogenesis by enhancing GABAergic support for the synaptic integration of postmitotic neurons. Moreover, 40 Hz flicker stimulation significantly improves spatial learning without causing adverse behavioral effects.

There are limitations to the current study. Our primary focus was to determine the mechanism of adult neurogenesis in response to long‐term light flicker. However, the specific neuronal circuitry that activates PV interneurons in response to 40 Hz light flicker remains unexplored. Additionally, the current study did not address the translational aspects of this technology, such as the optimal duration of treatment or potential adjunct therapies. Furthermore, the study was limited by its use of only male mice, and future research should consider the impact of sex, age, and disease models on the observed effects.

## 4 Experimental Section

### Experimental Animals

C57BL/6J mice were purchased from the Vital River Laboratory Animal Technology Co., Ltd. (Zhejiang, China). PV^Cre^ Mice (B6.129P2‐*Pvalb* ^1(cre)Arbr^/J) were purchased from Jackson Laboratory (Strain # 01 7320, USA) and bred locally. VIP‐Cre and CCK‐Cre mice were purchased from the Genepax Biotechnology Co., Ltd. (Guangzhou, China). To ablate DCX‐positive (DCX^+^) neurons, a knock‐in mouse model in which the diphtheria toxin (DT) receptor (DTR) was expressed under the control of the endogenous DCX promoter sequence via an internal ribosome entry site sequence was generated and used as previously described.^[^ ^28^, ^29^ ^]^ In these mice, DTR was expressed under the control of the DCX promoter, which allows for specific ablation of immature DCX‐expressing neurons after administration of DT while leaving the neural precursor pool intact. DCX^DTR^ knock‐in mice were bred on a C57BL/6J background and backcrossed for more than seven generations.

Male mice at the age of 6 months old were used for the study. Animals were maintained in a condition‐controlled room in a pathogen‐free SPFII animal facility (23 ± 1 °C, 50 ± 10% humidity). A 12:12 h light/dark cycle (7 a.m. to 7 p.m.) was automatically imposed, and the light intensity was maintained at 15–20 lx during the light period. However, the animal facility room light was at 200 lx during cleaning and experimental operation. Mice were housed in groups of six per individually ventilated cages and given access to food and water ad libitum. Experimenters were blinded to animals' treatments and sample processing throughout the subsequent experimentation and analyses.

### Ethical Approval and Animal Experimentation Design

Animal experiment protocols were approved by the Animal Care Committee of the Southern University of Science and Technology (SUSTech‐JY2018065‐A1). The ARRIVE guideline was followed when designing, performing, and reporting animal experimentation.^[^ ^71^ ^]^ Mice used in the current study were randomly assigned to each group to maintain total randomization.

According to the ARRIVE reporting guidelines, efforts were made to minimize the number of animals and animal suffering. The inclusion criterion was based on the pre‐established identical age and sex of the mice. Animal Experimentation Sample Size Calculator (AEEC) was used to determine the minimum sample size required to test the study hypothesis.^[^ ^72^ ^]^ Results indicated the required sample size to achieve 90% power for detecting 25% difference between two independent means at a significance criterion of *α* = 0.01 was n = 6. A minimum of 8 mice per group were used in the behavioral studies to achieve meaningful statistical differences. At least 3 mice per group were used for brain slice electrophysiology, immunostaining, fiber photometry recording, microdialysis, and local field potential recording. All experiments, including immunostaining, electrophysiology, and fiber photometry, as described in the manuscript, have been validated using separate batches of animals.

### Drug Administration

Saline was used to make 10 mg/ml 5′‐Bromo‐2′‐deoxyuridine (BrdU; B5002; Sigma) and 0.3 mg/ml clozapine N‐oxide (CNO; HY‐17366; MedChemExpress). The intraperitoneal (i.p.) administrations of BrdU (100 mg/kg) were carried out for the first four days of the last week of long‐term 40 Hz light flicker treatment and CNO (3 mg/kg) was carried out for 30 days during 40 Hz light flicker treatment. Diphtheria Toxin (DT; D0564; Sigma) was dissolved in phosphate‐buffered saline (PBS). The i.p. injection of DT (10 µg/kg) was carried out every 2 days for 2 weeks from the second week of 40 Hz light flicker treatment. Bicuculline (0.05 mg/ml, HY‐N0219, MCE) was dissolved in saline containing 1% DMSO and was injected i.p. at 0.5 mg/kg for 2 weeks during the 40 Hz light flicker treatment.

### Visual Stimulation and Audio Stimulation Protocol

The visual stimulation paradigm and methods were essentially as previously described.^[^ ^9^, ^10^ ^]^ The visual stimulation equipment, TangGuang, consisted of seven modules, including a tunable frequency signal generator and six LED lamps (36 V). The six LED lamps were interconnected in parallel circuits and evenly positioned around two transparent cages that housed the control and experimental groups simultaneously. Light stimulation was administered consistently once daily from 6 to 7 PM throughout the duration of the experiment, which lasted for up to 30 days.

For audio stimulation, two speakers were positioned above the transparent cages of the control and experimental groups, emitting a 10 kHz tone at a frequency of 40 Hz with a 50% duty cycle. The sound level experienced by the mice was approximately 60 dB.

### Active Place Avoidance (APA) Test

APA test was an effective, versatile, and repeatable tool to test hippocampus‐dependent spatial learning in rodents. The testing protocol was precisely as previously described.^[^ ^58^, ^73^, ^74^ ^]^ The mice were placed on a gridded platform with a 1‐meter diameter, enclosed by a clear Perspex cylinder (height: 32 cm; diameter: 77 cm) that rotated at 1 revolution per minute (Bio‐Signal Group, USA). A tracking computer defined a 60° shock zone, where any animal entering this region would receive a 0.5 mA foot shock after a 0.5‐second delay, with an inter‐shock delay of 1.5 seconds. Four visual cues were evenly distributed around the room, and the animal was consistently positioned in the arena opposite the shock zone for each day of testing.

The APA test protocol included one day of habituation without shocks for 5 minutes, followed by 5 days of testing with shocks administered for 10 minutes each day. The grid and platform were cleaned using 75% ethanol between testing trials. Parameters such as total distance traveled, number of shocks received, and number of entries into the shock zone were automatically recorded using a ceiling‐mounted video camera positioned overhead. The learning ability during the testing period was evaluated by comparing the parameters recorded on the 1^st^ day with those on the 5^th^ day of testing for each animal. The performance improvement was calculated as a percentage, reflecting the progress made during the task.

### Open Field Test

The open‐field test was utilized to assess the level of locomotion and anxiety in mice. The method used was as previously described.^[^ ^9^, ^10^ ^]^ Prior to the test, the mice were allowed to acclimate to the testing room for one hour. Each mouse was then placed in the center zone of the open field (40×40×40 cm). The open field was divided into 16 sections, with the four middle sections (20 cm x 20 cm) designated as the center area. The EthoVision XT software (Noldus Information Technology, Leesburg, USA) was used to record the total distance traveled by the mice and the amount of time spent in the center area during a 10‐minute period.

### Three Chamber Social Preference Test

The social behavior of mice was studied using a three chamber social preference test as previously described.^[^ ^10^ ^]^ The apparatus was divided into three interconnected chambers with transparent plexiglass. The mice were first habituated to the apparatus for 10 minutes. Sociability was then evaluated during a second 10‐minute period, during which the test mice could interact with either an empty cage or a genotype, age, and sex‐matched stranger mouse (Mouse 1) that was placed in a cage in one of the chambers. Preference for social novelty was then assayed in a third 10‐minute period by introducing a second stranger mouse (Mouse 2) into the previously empty cage. The time spent interacting with the empty cage, Mouse 1, or Mouse 2, was recorded, and the statistical range of 14 cm x 14 cm was measured using EthoVision XT 10 software. The sociability index (SI) and the social novelty preference index (SNI) were calculated as follows:

(1) $$\begin{array}{ccc} & & \mathrm{Sociability}\;\mathrm{index}\\ & & ≔\frac{interaction\;time\;with\;mouse1-interaction\;time\;with\;empty\;cage}{interaction\;time\;with\;mouse1+interaction\;time\;with\;empty\;cage} \end{array}$$

(2) $$\begin{array}{ccc} & & \mathrm{Social}\;\mathrm{novelty}\;\mathrm{preference}\;\mathrm{index}\\ & & ≔\frac{interaction\;time\;with\;mouse2-interaction\;time\;with\;mouse1}{interaction\;time\;with\;mouse2+interaction\;time\;with\;mouse1} \end{array}$$

### Elevated Plus Maze (EPM) Test

The EPM test was conducted in a white acrylic apparatus. The maze consisted of two open arms (50 × 10 cm) and two enclosed arms (50 × 10 × 40 cm) with open roofs. The arms intersected at a central square (10 × 10 cm). The maze was elevated 50 cm above the floor by a pedestal fixed beneath the central square. Mice were placed in the center of the maze, facing an open arm, at the start of the test and were allowed to explore for 5 minutes. The number of entries into the open arms and the total time spent in these arms were recorded as measures of anxiety. An entry was counted when all four paws of the mouse were inside the respective arm.

### Chemogenetic Activation or Inhibition of PV Interneurons

To selectively activate or inhibit PV interneurons, PV^Cre^ mice received a stereotactic injection of rAAV2/9‐hSyn‐DIO‐hM3D(Gq)‐eGFP‐WPRE‐pA (serotype 9, 120 nl) or rAAV2/9‐hSyn‐DIO‐hM4D(Gi)‐eGFP‐WPRE‐pA (serotype 9, 120 nl) into the bilateral DG hilus. The vector control AAV2/9‐hSyn‐DIO‐EGFP was utilized for comparison. All AAV viruses were procured from Taitool Bioscience Co. Ltd (Shanghai, China) and were diluted with PBS to a final concentration ranging between 5×10^12 and 1×10^13 genome copies per ml before stereotaxic administration into the mouse brain. Following a two‐week interval, mice were treated with 3 mg/kg of CNO via i.p. injection daily throughout the entire light flicker treatment period (30 days). Control mice received equivalent volumes of saline solution. Subsequently, the mice underwent APA test and neurogenesis analysis.

### Stereotaxic Surgery

Mice were anesthetized with isoflurane and then received bilateral injections of 200 nl of rVSVG‐Retrovirus‐CAG936‐eGFP‐3xFlag‐WRPE‐pA (concentration at 1.34E + 9 v.g./ml; v.g. viral genome). PV^Cre^ mice were also anesthetized with isoflurane and bilaterally injected with 200 nl of rAAV2/9‐CAG‐DIO‐taCaspase3‐TEVp‐WPRE‐pA (concentration at 3.3E + 12 v.g./ml) or rAAV2/9‐hSyn‐DIO‐hM3D(Gq)‐eGFP‐WPRE‐pA (concentration at 3.5E + 12 v.g/ml) or rAAV2/9‐hSyn‐DIO‐hM4D(Gi)‐eGFP‐WPRE‐pA (concentration at 3.3E + 12 v.g/ml) into the DG at a rate of 0.05 µl/min. The injection site was precisely located at AP: ‐2.0 mm, ML: ±1.5 mm, DV: ‐2.2 mm from bregma. After the injection, the needle was held in place for 5 minutes to allow for proper diffusion of the viral solution before being carefully withdrawn. The mice were then returned to their home cage to recover until fully awake. To minimize discomfort during the recovery period, meloxicam (1 mg/kg, s.c.) and penicillin (3000 U per mouse, i.p.) were administered once daily for three consecutive days.

### Brain Slice Electrophysiology

The protocol was modified from this previous studies.^[^ ^75^, ^76^ ^]^ Mice were anesthetized with 1% pentobarbital sodium and decapitated. The brains were quickly dissected and immersed in ice‐cold artificial cerebrospinal fluid (aCSF) containing (in mM): 30 NaCl, 26 NaHCO_3_, 10 D‐glucose, 4.5 KCl, 1.2 NaH₂PO_4_, 1 MgCl_2_, 194 sucrose, and 1.5 mL of 1 M HCl per liter of cutting solution. The solution was bubbled with 95% O_2_/5% CO_2_. Coronal brain slices (350 µm) were prepared using a vibratome (VT1120S, Leica Microsystems). The slices were allowed to recover for 30 min at 34 °C in aCSF containing (in mM): 124 NaCl, 26 NaHCO_3_, 10 D‐glucose, 4.5 KCl, 1.2 NaH_2_PO_4_, 1 MgCl_2_, 2 CaCl_2_, 10 g sucrose, and 1 mL of 1 M HCl per liter of aCSF, bubbled with 95% O_2_/5% CO_2_. After recovery, slices were transferred to a holding chamber at room temperature and allowed to stabilize for at least 1 hour before recording. For electrophysiological recordings, slices were placed in a recording chamber (RC26G, Warner Instruments, USA) on the x‐y stage of an upright microscope (BX51W, Olympus, Tokyo, Japan) and perfused with aCSF at a flow rate of 2 mL/min. All recordings were conducted at room temperature.

To measure sIPSCs and sEPSCs in DCX^+^ neurons and PV interneurons, patch clamp pipettes were filled with (in mM): 125 CsMeSO3, 5 NaCl, 10 HEPES (Na^+^ salt), 5 QX314, 1.1 EGTA, 4 ATP (Mg^2+^ salt), and 0.3 GTP (Na^+^ salt). sIPSCs were recorded in aCSF containing 50 µM D(‐)‐2‐amino‐5‐phosphonopentanoic acid (AP5) and 10 µM CNQX at a holding potential of +10 mV. The acquisition frequency was 20 kHz, and the filter was set to 2.9 kHz. For sEPSC recording, aCSF was supplemented with 20 µM (+)‐bicuculline at a holding potential of ‐60 mV. The acquisition frequency was also 20 kHz, with a 2.9 kHz filter.

For eEPSCs in PV interneurons, electrical stimulation (0.1 ms square pulse) was applied via a glass electrode filled with aCSF, positioned within 0.1 mm of the recording site. During the recording, aCSF containing 20 µM bicuculline was continuously perfused.

To assess the excitability of DCX^+^ neurons and PV, CCK, and VIP interneurons, patch pipettes were filled with (in mM): 128 potassium gluconate, 10 NaCl, 10 HEPES, 0.5 EGTA, 2 MgCl_2_, 4 Na_2_ATP, and 0.4 NaGTP. Current steps ranging from 0 pA to 380 pA (with a 20 pA increment and 1‐second duration) were applied. The increment was later adjusted to 5 pA to determine the rheobase current, defined as the minimum current required to elicit an action potential. The resting membrane potential was measured by injecting a 0 pA current. The acquisition frequency was set to 20 kHz, with a 2.9 kHz filter.

Data analysis was performed using Mini‐analysis (Synaptosoft Inc.) to evaluate the frequency and amplitude of sEPSCs and sIPSCs. Neuronal spiking was analyzed using Fitmaster software (HEKA Elektronik) to quantify the number of action potentials. Additionally, eEPSC amplitude was measured using Fitmaster software.

### Quantification of Neurogenesis by Immunofluorescent Staining

The immunostaining protocol was exactly as previously described.^[^ ^74^, ^77^ ^]^ Briefly, mice were administered phenobarbital sodium salt (0.1 g/kg) to induce anesthesia. Subsequently, the mice were subjected to transcardial perfusion with ice‐cold 0.01 M PBS, followed by 4% (weight/volume) paraformaldehyde (PFA) in 0.01 M PBS. Then the brains were retrieved and dehydrated in 15% sucrose for 1 day, and 30% sucrose for 2 days. Serial coronal brain sections (40 µm thickness) through the hippocampus were cut and retained for immunostaining. A total of 72 consecutive slices from the beginning of the DG (at AP axis = ‐1.4 mm from bregma, coronal view) were collected and then preserved in cryo‐protectant solution at ‐20 °C. One‐sixth of each brain was pooled into one series for immunohistochemistry.

Brain slices were retrieved from cryo‐protectant solution and washed a float 3 times in PBST before staining. The slices were transferred onto glass slides and incubated in 0.5% Triton X‐100 at 37 °C for 30 min. After permeabilization and washing with PBST, the slices were heated for 2 minutes in citrate buffer (pH = 6.0) with an autoclave (105 °C) for antigen retrieval. After retrieval, the slices were cooled down in citrate buffer to room temperature, followed by PBST washing. For BrdU immunostaining, sections were treated with pre‐warmed 1 M HCl for 30 min at 37 °C to disrupt hydrogen bonds between bases and to denature the DNA. These slices were then blocked in 10% goat serum PBS with 0.2% TritonX‐100 at room temperature for 1 h. The primary antibody binding was carried out in 3% goat serum PBST solution with 0.2% Triton X‐100 at 4 °C overnight. The following primary antibodies were used: anti‐BrdU (Abcam, rat, 1:500), anti‐DCX (Abcam, rabbit, 1:500), anti‐GABA (Sigma, rabbit, 1:500), anti‐GAD67 (Abcam, rabbit, 1:500), anti‐Tbr2 (Abcam, rabbit, 1:500), and anti‐PV (Abcam, rabbit, 1:500). On the second day, the slices were washed with PBST 3 times and incubated with the secondary antibody at room temperature for 1 h. After washing, the slides were observed using a Zeiss LSM980 confocal microscope.

The methodology employed to quantitatively assess the quantities of DCX and BrdU‐positive cells for neurogenesis was as outlined in a previous study. Positive cells were counted on every sixth section (240 µm apart) across the entire dorsal‐ventral span of the DG. The counts were then multiplied by six to determine the total number of labeled cells within the DG of each brain.

### Analysis of PV^+^ Perisomatic Puncta on DCX^+^ Cells

Images were captured using a Zeiss LSM980 confocal microscope equipped with a 63× lens. 3–4 sections were obtained from each mouse for analysis. To analyze perisomatic puncta in the images, the cell soma profile was manually outlined in ImageJ software, and a series of custom‐made macros in Fiji were used, as previously described. To define the region of interest (ROI), the initial outline was expanded by 1 µm from the cell body's surface. The ROI was established as the enclosed area between these two outlines. Subsequently, the total number of puncta within the ROI was counted, and the perimeter of the cell soma was calculated. The density of PV perisomatic puncta was determined by dividing the number of puncta by the perimeter.

### Fiber Photometry Recording and Analysis

The fiber photometry method was performed as previously described.^[^ ^78^ ^]^ Mice were anesthetized with isoflurane and received a unilateral injection of 250 nl of rAAV2/9‐hSyn‐DIO‐jGCaMP7f‐WPRE‐pA (Taitool, China), 200 nl of AAV2/9‐hSyn‐iGABASnFR‐WPRE‐pA (Brain Case, China), or 200 nl of rAAV2/9‐hSyn‐GRABeen ACh3.0‐WPRE‐pA (Taitool, China) into the DG at a rate of 0.05 µl/min. The stereotactic injection site was targeted at AP: ‐2.0 mm, ML: ±1.5 mm, and DV: ‐2.2 mm from bregma. The needle was left in place for 5 minutes post‐injection before being removed. Afterward, the mice were allowed to recover in their home cages until fully awake. To alleviate post‐operative pain and infection, meloxicam (1 mg/kg, s.c.) and penicillin (3000 U per mouse, i.p.) were administered once daily for 3 days.

After a three‐week period, a 1.25‐mm‐diameter optical fiber was implanted at a depth of 1.75 mm to capture the fluorescence signals emitted by the calcium or neurotransmitter sensor. The fiber was attached to the implanted ferrule using a ceramic sleeve. A commercial two‐channel fiber photometry system (Thinker Tech Nanjing Biotech, China) was utilized to record the emissions. This system employs a 405 nm LED as the isosbestic control channel and a 470 nm LED for excitation. Recordings were conducted on freely moving mice in a square enclosure (sides: 40 cm) during light flicker visual stimulation.

Initial processing of the fiber photometry data was performed using a custom MATLAB script based on the pMat application from BarkerLab (https://github.com/djamesbarker/pMAT) to handle raw data from individual trials. Fluorescence signal processing and analysis followed a previously described comprehensive guide on fiber photometry studies.^[^ ^78^ ^]^ First, the data from signal channel (470 nm) and isosbestic control channel (405 nm) were extracted. Second, the channels were smoothed utilizing Lowess, a local linear regression method. Then scale of channels was normalized using the MATLAB *polyfit* function. In other words, the raw signals were corrected using isosbestic control signals to account for any photo‐bleaching effects:

(3) $$a\left(slope\right)=\frac{N\sum \left(xy\right)-\sum x\sum y}{N\sum \left(x^{2}\right)-\sum {\left(x\right)}^{2}}$$

(4) $$b\left(intercept\right)=\frac{\sum y-m\sum x}{N}$$

N was the number of subjects, x was the control channel, and y was the signal channel. the resulting slope and intercept were then used to generate a scaled control channel, (MATLAB figure titled “Signal versus Fitted Control”):

(5) ScaledControlChannel=a∗ControlChannel+b

Subsequently, the delta F/F (ΔF/F) was generated by subtracting the fitted control channel from the signal channel:

(6) $$\frac{\Delta F}{F}=\frac{Signal\;Channel-Scaled\;Control\;Channel}{Scaled\;Control\;Channel}$$

Finally, the signals were transformed into robust z‐scores using the following formula:

(7) $$\begin{array}{c} \mathrm{Robust}\;Z\;\mathrm{Score}\;\left(i\right)=\frac{\Delta F/\mathrm{FEve}\mathrm{nt}\left(i\right)-\mathrm{median}\left(\Delta F/\mathrm{Fbas}\mathrm{eline}\right)}{\mathrm{median}\mathrm{absolute}\;\mathrm{deviation}\;\left(\mathrm{MAD}\right)\;\mathrm{of}\;\mathrm{baseline}} \end{array}$$

To assess neuronal responses, Peri‐event time histograms/heatmaps (PETHs) were used to examine the stability of a response over repeated trials. An event window with a 5‐second length was set around each event, including 5 seconds before the event and ends 5 seconds after the event. The final Z scores were generated using an event window and an additional baseline window.

### Microdialysis and HPLC Analysis

The procedures were conducted exactly as previously described.^[^ ^79^ ^]^ An MBR intracerebral guide cannula (MD‐2255, BASI) was surgically implanted into the DG of the mice at the following coordinates: AP: ‐2.0 mm, ML: ±1.5 mm, DV: ‐2.2 mm from bregma. Prior to the experiment, the mice were anesthetized, and an MBR‐1‐5 brain microdialysis probe (MD‐2211, BASI) was inserted into the cannula. aCSF was prepared with the following components (in mM): 124 NaCl, 26 NaHCO_3_, 10 D‐glucose, 4.5 KCl, 1.2 NaH_2_PO_4_, 1 MgCl_2_, and 2 CaCl_2_, supplemented with 10 g of sucrose and 1 mL of 1 M HCl per liter of aCSF, and bubbled with 95% O_2_/5% CO_2_. This solution was infused into the microdialysis system at a constant rate of 1 µl/min using a syringe pump. Dialysates were continuously collected for 45 minutes under stable lighting conditions. Following this, after 1 hour of exposure to 40 Hz light flicker, the collection period was extended for 60 minutes, with dialysates collected every 15 minutes while the mice remained under anesthesia. Here, the 15‐minute interval between collections was crucial as it ensures that enough time has passed to allow the neurotransmitters and other molecules to accumulate in the brain's interstitial fluid to reach a detectable baseline concentration necessary for accurate sample analysis. This systematic approach guarantees that any observed changes in the dialysate composition can be attributed to the experimental conditions rather than fluctuations due to insufficient sampling time. The concentration of GABA in the dialysates was subsequently quantified using HPLC analysis as previously described.

### Recordings of Local Field Potential (LFP)

The procedures used were as precisely described.^[^ ^9^ ^]^

### Implantation of Electrodes

Mice were anesthetized with a mixture of 2.5% isoflurane in N_2_:O_2_ (70:30; flow rate 400 ml/min) in an induction chamber. Anesthesia was maintained with 1.5% isoflurane in N_2_:O_2_ mixture during surgery using an isoflurane vaporizer (#R540, RWD Life Science, Shenzhen, China). The animal's body temperature was maintained at 37.0 ± 0.5 °C using a rectal temperature probe and a heating pad (#TCAT‐2DF, Harvard Apparatus, USA).

A cranial window at 1.2 mm in diameter with AP: ‐2.0 mm, ML: ±1.5 mm, DV: ‐2.2 mm from bregma over the DG was created using a dental drill (#78 001, RWD Life Science) guided by stereotaxis (# 68 861 N, RWD Life Science). A 4‐channel microwire array electrode (35 µm, Stablohm 650, California Fine Wire Co., USA) was inserted into DG and immobilized using kwiksil (Item #KWIK‐SIL, Microprobes for Life Science, Gaithersburg, MD, USA). The tetrode was anchored to the skull bone using four skull screws (1.0 mm diameter) and embedded in dental cement. Mice were moved into a warm (37.0 ± 1 °C) recovery chamber (#DW‐1, Harvard Apparatus) for 1 h.

After a 4‐day recovery period following electrode implantation surgery, the mice were acclimated to the recording setup by being placed in a box measuring 50 cm in depth, 50 cm in width, and 50 cm in length for 10 minutes per day until the first recording session. Prior to each recording session, the box was sanitized using 75% ethanol. During the recording session, a helium balloon delicately supported the implant, enabling the mouse to move freely within the box.

The head‐stage was connected to the OmniPlex Neural Recording Data Acquisition System (Plexon Inc., Dallas, TX). A camera was positioned above the box to record the behavioral states of the mouse. An LED lamp, generating flicker lights at specific frequencies, was placed at the front of the box. Local field potentials (LFPs) were recorded for a minimum of 20 minutes per mouse. The LFP signals were sampled at 1000 Hz with a band‐pass filter set between 0.5 and 120 Hz. Raw data were stored for subsequent offline analysis. Statistical analysis was performed using data from 5 mice per group.

To identify the most relevant epochs of LFPs for analysis, the relationship between mouse movement behavior and LFPs using simultaneous video recordings of movement and LFPs recording data was investigated. LFPs provide valuable insights into neural activity across various behavioral states, with changes in LFP patterns closely linked to specific movements and motor behaviors of the mouse. For example, LFPs typically show an increase in theta rhythm during voluntary movement or exploration, particularly during running or active navigation. Furthermore, hippocampal theta rhythm was strongly associated with movement velocity, with faster movements often correlating with higher‐frequency theta oscillations.^[^ ^9^ ^]^ Based on these considerations, several 5‐second epochs of LFPs recorded during the mouse resting state (stationary) was selected, as determined by the video recordings, for analysis in order to eliminate potential interference from movement‐related alterations in the LFPs.

A multitaper fast Fourier transform method was implemented for power spectral analyses using MATLAB (ver 9.11.0, R2021b) software. Data was filtered with a band‐pass filter of 0.1 to 100 Hz and a notch filter of 50 Hz. The power spectrum in Figure 4 was given by a multitaper estimation method using MATLAB using Equation (8). Given a time series *X_n_*, *n*  =  1, 2, …, *N*, the number of the Slepian sequences was *K  =  2NW−1*. The simplest multitaper estimate of the spectrum was given by

(8) $$S\;\left(f\right)=\frac{1}{K}\;\sum_{k=1}^{N}{\left|\;\frac{1}{N}\;\sum_{n=1}^{N}\exp\left(2\pi ifn\right)\;u_{n}^{k}\;X_{n}\right|}^{2}$$

where $u_{n}^{k}$, n = 1, 2,…, N was the *k*th Slepian sequence.

The Short‐time Fourier transform (STFT) was applied to reveal the power changes in different frequencies on a time scale. The LFP data was segmented into epochs of the same time interval as described above, and a hamming window with a length of 8000, 50% of overlap was used to calculate the spectral amplitude between 1 and 100 Hz through a MATLAB (Ver 9.11.0, R2021b) function *stft*.

### Insertion of GRIN Lens in Hippocampus DG

The mice were anesthetized using a gas anesthesia machine (RWD Life Science) and positioned carefully on a stereotaxic apparatus. A stereoscope was adjusted to provide a clear view of the skull's surface. Eye ointment (Guangzhou Pharmaceutical Holdings, China) was applied, and a heating pad set to 35 °C (Physitemp, USA) was used to maintain body temperature. The hair and scalp were then carefully removed with sterilized scissors, and subcutaneous tissue was gently scraped away with a cotton swab.

To ensure precise alignment, the heights of bregma and lambda were adjusted along with lateral measurements. Using a cranial drill (RWD Life Science), two 1.0 mm diameter holes were created in the frontal bone, stabilized with cranial pins from the same manufacturer. With bregma as the reference point, coordinates were set at ML: ‐1.5 mm, AP: ‐2 mm. A 1.2 mm diameter hole was drilled at this location, and the skull fragment was carefully removed. Aspiration was performed to expose the hippocampal fibrous capsule and molecular layer, with additional aspiration done as needed. Any bleeding was controlled using a hemostatic sponge (Fukangsen Medical Device, Guilin, China).

The GRIN lens was carefully inserted into the hole while the nVoke imaging system (Inscopix Inc., USA) was activated, ensuring the lens bottom was visible. The lens was secured in place with bio‐compatible silicone gel (WPI Surgical Instruments, USA), and a titanium ring was affixed around the lens using acrylate adhesive. Dental cement (Shanghai Yuyan Instruments, China) was applied to cover the exposed skull areas, with a drop of silicone gel placed on the lens surface for additional protection against damage.

### Microvasculature Imaging Using Two‐Photon Microscopy

Mice equipped with the GRIN lens were anesthetized using a gas anesthesia machine (RWD Life Science). Their hindlimbs were secured with adhesive tape, and fur was removed using depilatory cream. The skin was then sanitized with iodine tincture. After making an incision in the skin, a 200 µl mixture containing 150 µl of 70 kDa Dextran‐FITC and 50 µl of Hydrazide‐Alexafluor 647 (Thermo Fisher, USA) was slowly injected into the exposed femoral vein using an insulin syringe to stain blood vessels and arterioles, respectively.^[^ ^80^ ^]^ Hemostasis was achieved with a hemostatic sponge, and the skin was closed with sutures (Shanghai Jinhuan Medical, China). The silicone gel previously applied to the top of the GRIN lens was carefully removed with forceps. The cranial window or lens surface was gently cleaned using an alcohol‐soaked hemostatic sponge. The mice were then secured onto a custom‐made platform via the implanted titanium ring, ensuring proper alignment under the imaging objective. Imaging began once the mice had fully regained consciousness.

An upright FVMPE‐RS multiphoton microscopy system (Olympus, Japan) was used, featuring an Olympus upright microscope with a water immersion objective (25X/1.05, W.D. = 2 mm). A femtosecond‐pulsed Ti:Sa laser (Mai Tai DeepSee, Spectra‐Physics, USA) was employed to generate a 1000 nm excitation wavelength for both FITC and Alexa Fluor 647. The laser power was set to 15% of 1.28 W. Two acquisition channels were used: FV30‐FGR (495–540 nm) for FITC and FV30‐FRCY5 (660–750 nm) for Alexa Fluor 647. Imaging parameters were as follows: a 512×512 pixel format, digital magnification of 3X (resulting in a pixel size of 0.931 µm/pixel), and Galvo unidirectional scanning mode.

The module's position under the objective was adjusted to ensure the image was clearly visible in the field of view. Imaging was performed with 40 Hz light flicker. Imaging of the DG was repeated on the following day without light flicker. To reduce image noise from 40 Hz light flicker, an aluminum foil was attached to the mice's head, separating the lens and eyes. A custom 40 Hz LED light flicker module with a light‐conducting tube focused light on the eyeball during imaging.

Hydrazide fluorescence captured through the CY5 channel marked the arteriole lumen.^[^ ^80^ ^]^ The transition point, where the hydrazide signal ended along the vasculature, was identified as the boundary between the arteriole and capillary. The capillaries were categorized as follows: the first to fourth branched capillaries from the transition point were designated as pre‐capillary, and the fifth to eighth as post‐capillary. The terminal arteriole was defined as the last segment of the arteriole with a hydrazide signal, located immediately before the transition point. The FITC signal captured through the FGR channel represented the flow of blood cells within the vessels. Each vessel was imaged to assess its morphology and length. Three‐line scans were performed by aligning the scanning line along the direction of vessel extension, with a scan time limited to 1 second. Between 3 and 8 vessels from each vessel category (arteriole, pre‐capillary, and post‐capillary) were randomly selected and imaged across the entire field of view. After imaging, the mice were carefully removed from the module and returned to their rearing cage. Data were then collected for subsequent analysis.

### Statistical Analysis

Data were presented as the mean ± standard error of the mean (SEM). Statistical analyses were performed using Prism (V10, GraphPad Software, La Jolla, CA, USA). Normality of data distribution was assessed using the Shapiro‐Wilk test before selecting the appropriate parametric or non‐parametric test. For two‐group comparisons, an unpaired t‐test was used if the data were normally distributed; otherwise, the Mann‐Whitney U test was applied. For comparisons among multiple groups, one‐way or two‐way ANOVA with Tukey's *post hoc* test was used if the data passed the normality test; otherwise, the Kruskal‐Wallis test with Dunn's *post hoc* test was applied. Firing rate data were analyzed using two‐way repeated measures (RM) ANOVA with either Tukey's or Sidak's *post hoc* test. Specific statistical details, including sample size (n), precision measures, tests used, and significance definitions, were provided in the figure legends. A P‐value of <0.05 was considered statistically significant.

## Conflict of Interest

The authors declare no conflict of interest.

## Author Contributions

H.Y., Y.W., X.D., S.W. contributed equally to this work. H.Y., X.D., S.W., Y.W., and M.Y., did the behavior tests. H.Y., X.D., S.W., Y.W., J.M., Y.P., J.C., and S.C. did immunostaining assays, light and sound stimulation treatments. J.J., Y.P., Y.W., and J.C. performed electrophysiological experiments. S.W., B.L., H.W. and Z.Z. did the local field potential recording and analysis. J.D. and S.W. did the microdialysis sample collection, and J.D. did HPLC analysis. S.W. did the fiber photometry recording and analysis. P.B. and T.W. provided the DCX inducible deletion mice. S.‐T. H., J.J., and Y.W. designed the project, analyzed data, supervised the project and wrote the manuscript. All authors contributed to the article and approved the submitted version.

## Supporting information



Supporting Information

## Data Availability

The data that support the findings of this study are available from the corresponding author upon reasonable request.
