# Supplementary material for: Parvalbumin Interneuron‐Dependent Hippocampal Neurogenesis Evoked by Prolonged Rhythmic Light Flicker
Source: Adv Sci (Weinh). 2025 Jun 30;12(37):e03017. doi: 10.1002/advs.202503017 (PMC12499440; doi:10.1002/advs.202503017)
Supplement: Supplementary file 1 — Supporting Information [file ADVS-12-e03017-s001.docx]

**Supplemental Figures**

**Parvalbumin Interneuron-dependent Hippocampal Neurogenesis Evoked by Prolonged Rhythmic Light Flicker**

Hai Yan^1,#^, Yunxuan Wang^1,#^, Xufan Deng^1,#,a^, Shiyu Wu^1,#^, Yifan Pan^1^, Jun Du^2^, Mei Yu^1,b^, Bo Liu^1^, Huimei Wang^1,c^, Zhengyu Zhang^1,4^, Jinghong Chen^1^, Shuifa Chen^1^, Yizheng Wang^3^, Tara Walker^4^, Perry Bartlett^4^, Jun Ju^1*^, Sheng-Tao Hou^1,*^

^1^ Brain Research Centre, Department of Neurobiology, School of Life Sciences, Southern University of Science and Technology, 1088 Xueyuan Blvd, Shenzhen, Guangdong Province, 518055, P. R. China.

^2^ The Brain Science Centre, Beijing Institute of Basic Medical Sciences, Beijing, 100850, China.

^3^ Huashan Hospital, Fudan University, Shanghai, P. R. China.

^4^ Clem Jones Centre for Ageing Dementia Research, Queensland Brain Institute, The University of Queensland, Brisbane, Australia.

Present addresses:

^a^ University of Tübingen, Geschwister-Scholl-Platz, 72074 Tübingen Germany

^b^ Shenzhen Bay Laboratory, Shenzhen, Guangdong 518107, China

^c^ Department of Anatomy and Neurobiology, College of Medicine, Northeast Ohio Medical University, Rootstown, USA.

^#^These authors contributed equally

*Prof. Sheng-Tao Hou, Dr. Jun Ju

**Email:** [hou.st@sustech.edu.cn,](mailto:hou.st@sustech.edu.cn,) [juj@sustech.edu.cn](mailto:juj@sustech.edu.cn)

**
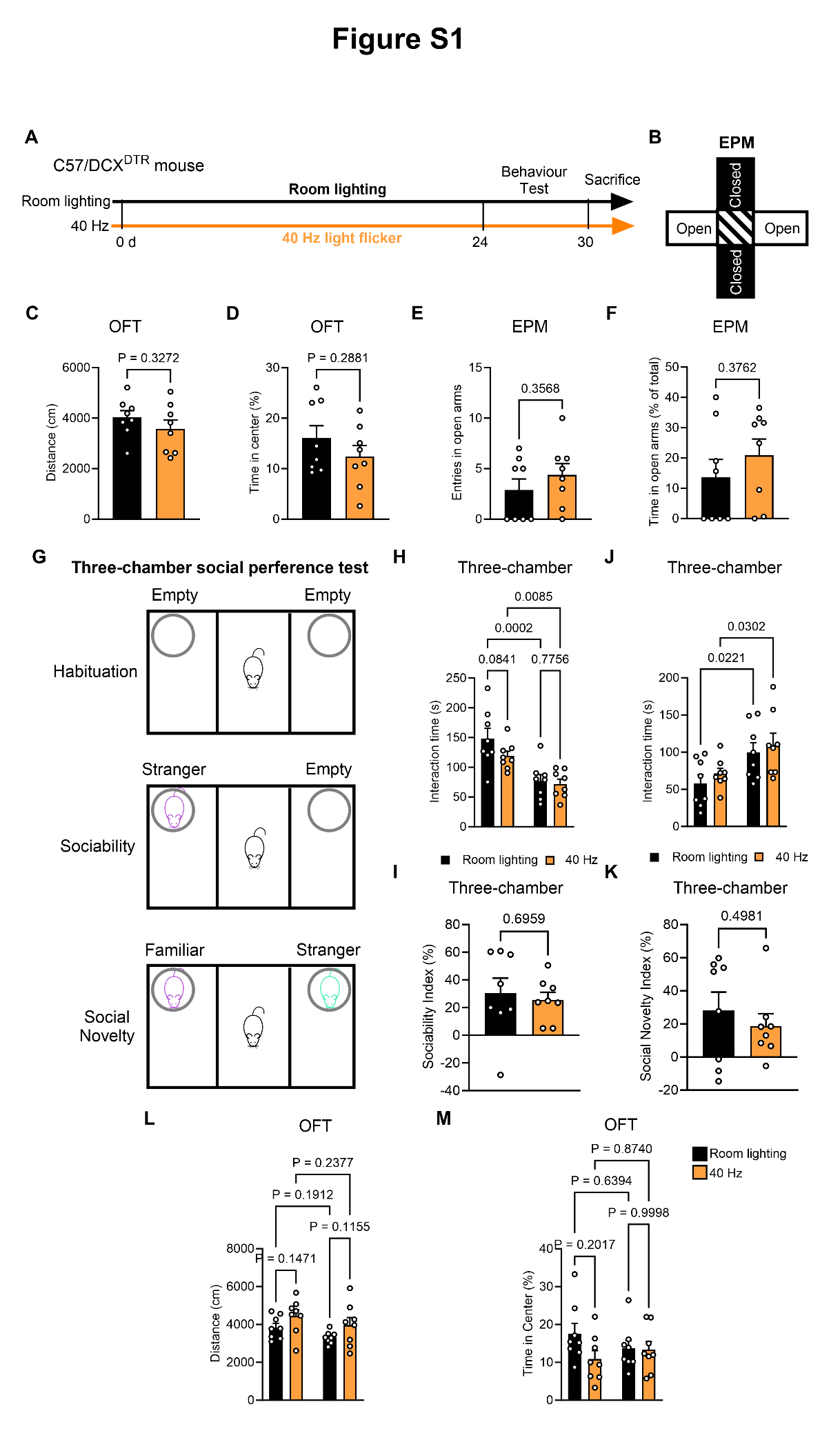
**

**Figure S1.** Long-term 40 Hz light flicker does not alter motor and social behaviors. A) Experimental schema for light flicker treatment (orange colored line) and behavior tests. B) A diagram of the elevated plus maze (EPM) test. C, D) Total distance traveled (C) and time spent in the center (D) for the open-field test (OFT) [two-tailed unpaired t test for C: t_(14)_ = 1.015, P = 0.3272; and for D: t_(14)_ = 1.104, P = 0.2881]. n = 8 mice per group. E, F) Measurements for entries in open arms (E) and time spent in open arms (f) for the EPM test [two-tailed unpaired t test for E: t_(14)_ = 0.9529, P = 0.3568; and for f: t_(14)_ = 0.9139, P = 0.3762]. G) A diagram of the three-chamber social preference test, which includes three phases: habituation, sociability, and social novelty. H-K) No significant difference in interaction time between No flicker and 40 Hz group in sociability phase (H) and social novelty phase (J). There was also no significant difference in sociability index (I) and social novelty index (K) between the two groups. L, M) The total distance traveled (L) and time spent in the center (M) in OFT were measured and compared between the four groups of DCX^DTR^ mice. *n* = 8 mice per group. Student's t test was performed for panels I, K and a two-way ANOVA with Tukey's *post hoc* test was performed for panels H, J, L, M. Specific P values were indicated in the panels.

**
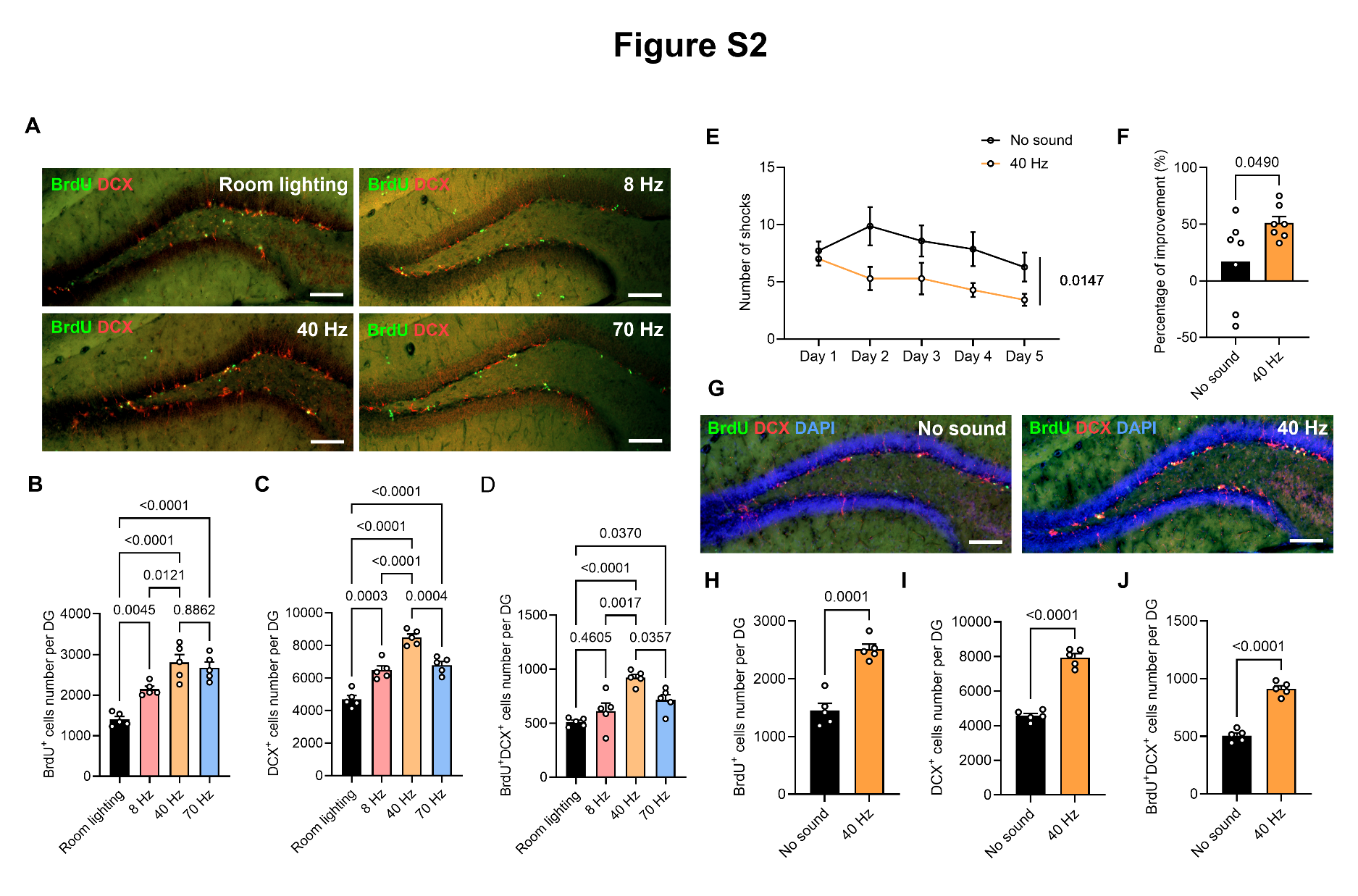
**

**Figure S2.** Neurogenesis evoked by long-term treatment with different frequencies of light flicker and 40 Hz auditory stimulus. A) Double immunostaining for BrdU (green) and DCX (red) in the DG region of four groups of mice. Scale bars, 100 μm. B-D) 40 Hz and 70 Hz significantly increased the number of BrdU^+^ cells (B), DCX^+^ cells (C), and BrdU^+^/DCX^+^ cells (D) in the DG, while 8 Hz group only increased the number of BrdU^+^ cells and DCX^+^ cells. *n* = 5 mice per group. E) Adult mice were subjected to 1 hour daily for 30 days of 40 Hz audio stimulation. The 40 Hz group received significantly fewer shocks compared to the No sound control mice in the APA test [two-way RM ANOVA with Tukey's *post hoc* test with F_(1, 12)_ = 8.113, P = 0.0147 showing the effect of flicker treatment on the shocks of APA test]. *n* = 7 mice per group. F) The percentage improvement in avoiding shocks on the 5^th^ day of the APA test compared to the 1^st^ day of the APA test [two-tailed unpaired t test with t_(12)_ = 2.190, P = 0.0490]. G) Double immunostaining showing BrdU (green), DCX (red) with DAPI (blue) in the DG of No sound and 40 Hz sound treated mice. Scale bars, 100 μm. H-J) Long-term 40 Hz sound treatment significantly increased the number of BrdU^+^ cells (H), DCX^+^ cells (I), and BrdU^+^/DCX^+^ cells (J) in the DG. Student's t test was performed for panels H-J, with specific P values as indicated. *n* = 5 mice per group.

**
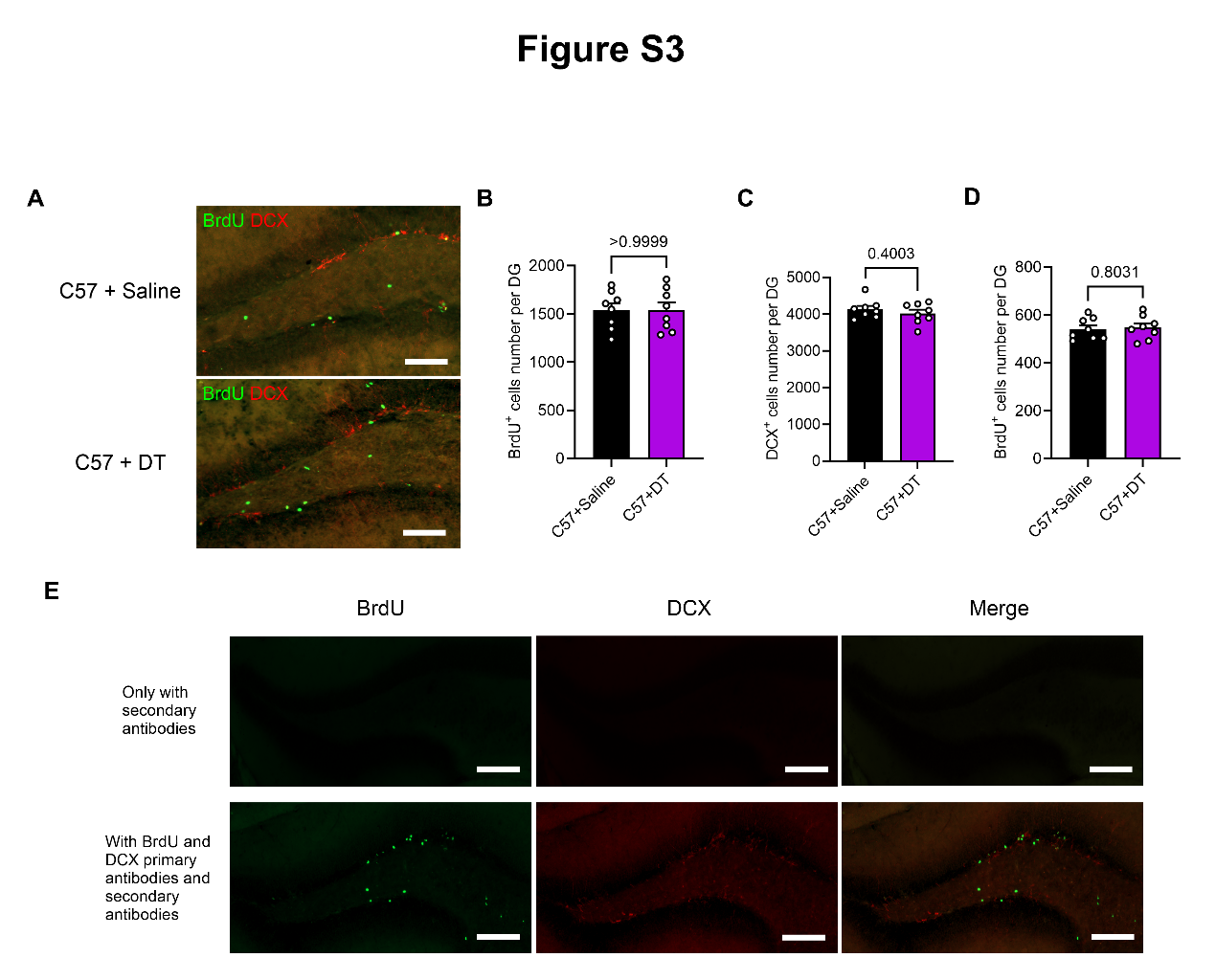
**

**Figure S3.** Effects of DT administration on neurogenesis in wild-type mice. A) Double immunostaining showing BrdU (green), DCX (red) in the DG of C57+Saline and C57+DT mice. Scale bars, 100 μm. B) Quantification of the number of BrdU^+^ cells in the DG [two-tailed unpaired t test: t_(14)_ = 0.000, P > 0.9999]. C) Quantification of the number of DCX^+^ cells in the DG [two-tailed unpaired t test: t_(14)_ = 0.8675, P = 0.4003]. D) Quantification of the number of BrdU^+^/DCX^+^ cells in the DG [two-tailed unpaired t test: t_(14)_ = 0.2541, P = 0.8031]. *n* = 8 mice per group. E) Negative control of BrdU and DCX immunostaining without primary antibodies. Scale bars, 100 μm.

**
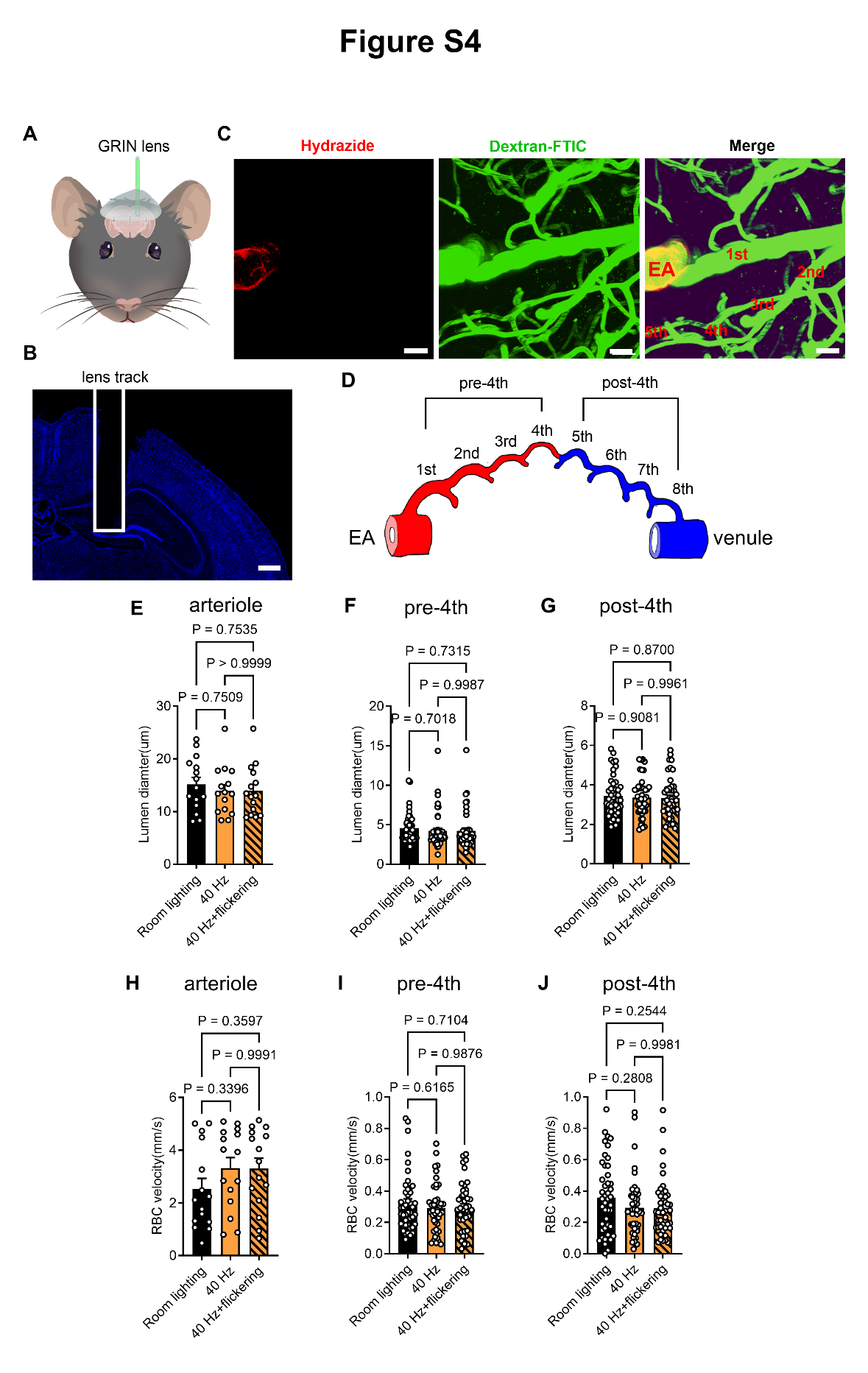
**

**Figure S4.** Long-term 40 Hz light flicker does not alter DG regional blood flow. A) A diagram of GRIN lens insertion in the hippocampus. B) Brain section indicates the location of the GRIN lens in the DG molecular layer (white-colored box). C) Two-photon live imaging of Alexa Fluor™ 647 Hydrazide labeled arteriole (red color) and Dextran-FITC labeled arterioles and capillaries (green color). There were 3 groups: Room lighting (control), 40 Hz (30 days of 40 Hz light flicker treatment without flickering at the time of imaging), and 40 Hz+flickering (30 days of 40 Hz light flicker treatment plus 40 Hz flickering at the time of imaging). D) A diagram depicting branching capillaries from the arteriole (EA) to the venule. E-G) Quantification of alterations of lumen diameter during 40 Hz light flicker as measured using two-photon microscopy. H-J) Quantification of the red blood cell velocity. One-way ANOVA with Tukey's *post hoc* test was performed for panels from E-J. Scale bar, 10 µm. *n* = 105 vessels from 3 mice per group. Specific P values were as indicated.


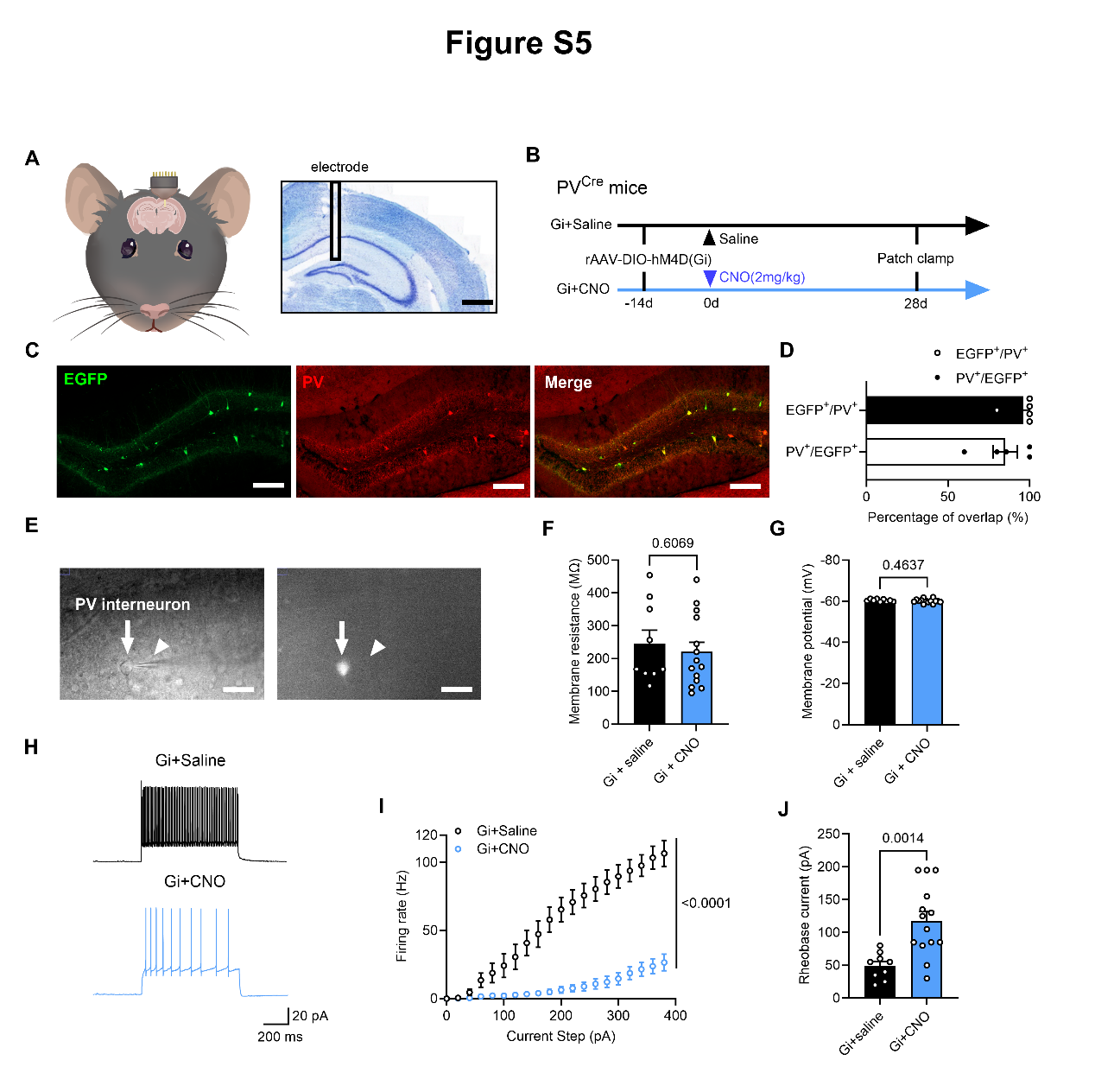


**Figure S5.** Effects of chemogenetic inactivation of PV interneurons. A) An image of the brain coronal section of the left hippocampus showing the LFP recording track in the DG. B) Experimental design showing two groups of PV^Cre^ mice injecting with AAV-DIO-hM4D (Gi) virus (Gi+Saline group and Gi+CNO group). C) Double immunostaining for Gi-EGFP (green) and PV (red) of the DG. Scale bar, 20 μm. D) Determination of percentages of PV^+^ cells expressing Gi-EGFP (EGFP^+^/PV^+^) and Gi-EGFP^+^ cells expressing PV (PV^+^/EGFP^+^). *n* = 15 brain slices from 5 mice. E) Images of fluorescent positive PV interneurons close to DG granule layer identified using a fluorescence microscope and phase contrast bright field for patch clamp recording. Arrowheads indicate the glass recording pipette, while arrows indicate the PV interneuron. Scale bars, 20 μm. F) Quantification of membrane resistance R_m_ of PV interneurons from the Gi+Saline and Gi+CNO groups [two-tailed unpaired t test with t_(21)_ = 0.5224, P = 0.6069]. G) Quantification of resting membrane potential of PV interneurons from the Gi+Saline and Gi+CNO groups [two-tailed unpaired t test with t_(21)_ = 0.7464, P = 0.4637]. H) Representative firing rate traces recorded in PV interneurons from the Gi+Saline and Gi+CNO groups. I) Quantification of the firing rate of PV interneurons [two-way RM ANOVA with Tukey's *post hoc* test to analyze the effect of the CNO treatment and the current injected on the neuron. There was a statistical significance of CNO treatment on firing rate with F_(21, 399)_ = 28.80, P <0.0001]. J) Quantification of the rheobase current [two-tailed unpaired t test with t_(21)_ = 3.691, P = 0.0014]. Gi+saline group: *n* = 4 mice with 9 cells recorded. Gi+CNO group: *n* = 4 mice with 14 cells recorded.


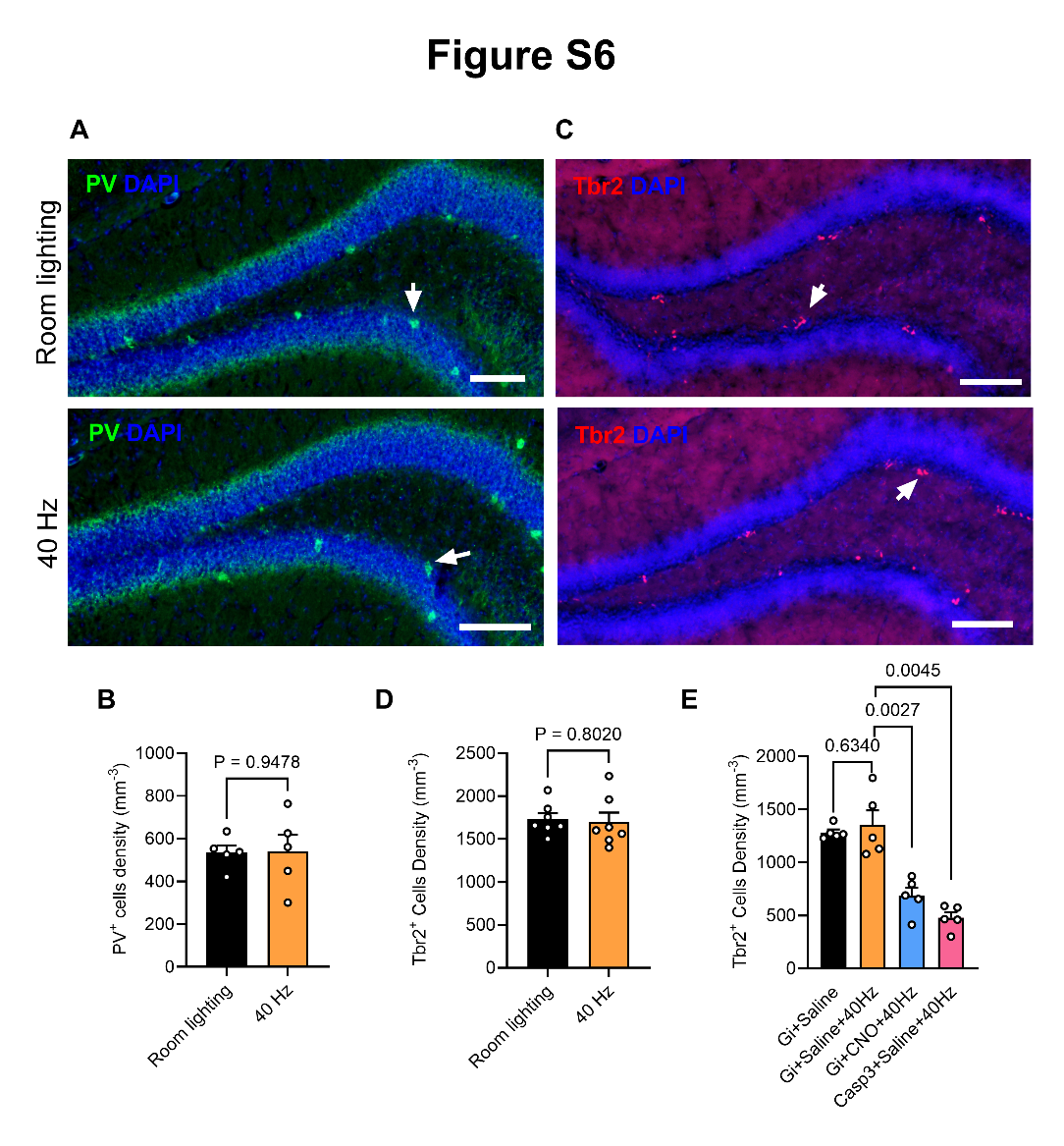


**Figure S6.** Prolonged exposure to 40 Hz light flicker does not affect the density of PV^+^ and Tbr2^+^ cells in DG. A) Images of immunostaining of hippocampal sections showing PV interneurons (A, arrows). B) Quantification of PV density after long-term 40 Hz light flicker treatment [two-tailed unpaired t test with t_(6)_ = 0.2421, P = 0.8167]. *n* = 24 brain sections from 4 mice per group. C) Images of immunostaining of hippocampal sections showing Tbr2^+^ cells (C, arrows) post long-term 40 Hz light flicker. D) Quantification of Tbr2^+^ cell density [two-tailed unpaired t test with t_(12)_ = 0.2563, P = 0.8020]. Scale bar, 100 µm. *n* = 42 brain sections from 7 mice per group. E) The number of Tbr2^+^ cells was significantly reduced in PV-inactivated (Gi+CNO+40Hz group) and PV-deleted (Casp3+Saline+40Hz group) mice. The Students' t-test was performed for panel E with P values as indicated. *n* = 18 brain sections from 3 mice of Gi+Saline and Casp3+Saline+40 Hz groups; *n* = 30 brain sections from 5 mice of Gi+Saline+40 Hz and Gi+CNO+40 Hz group.


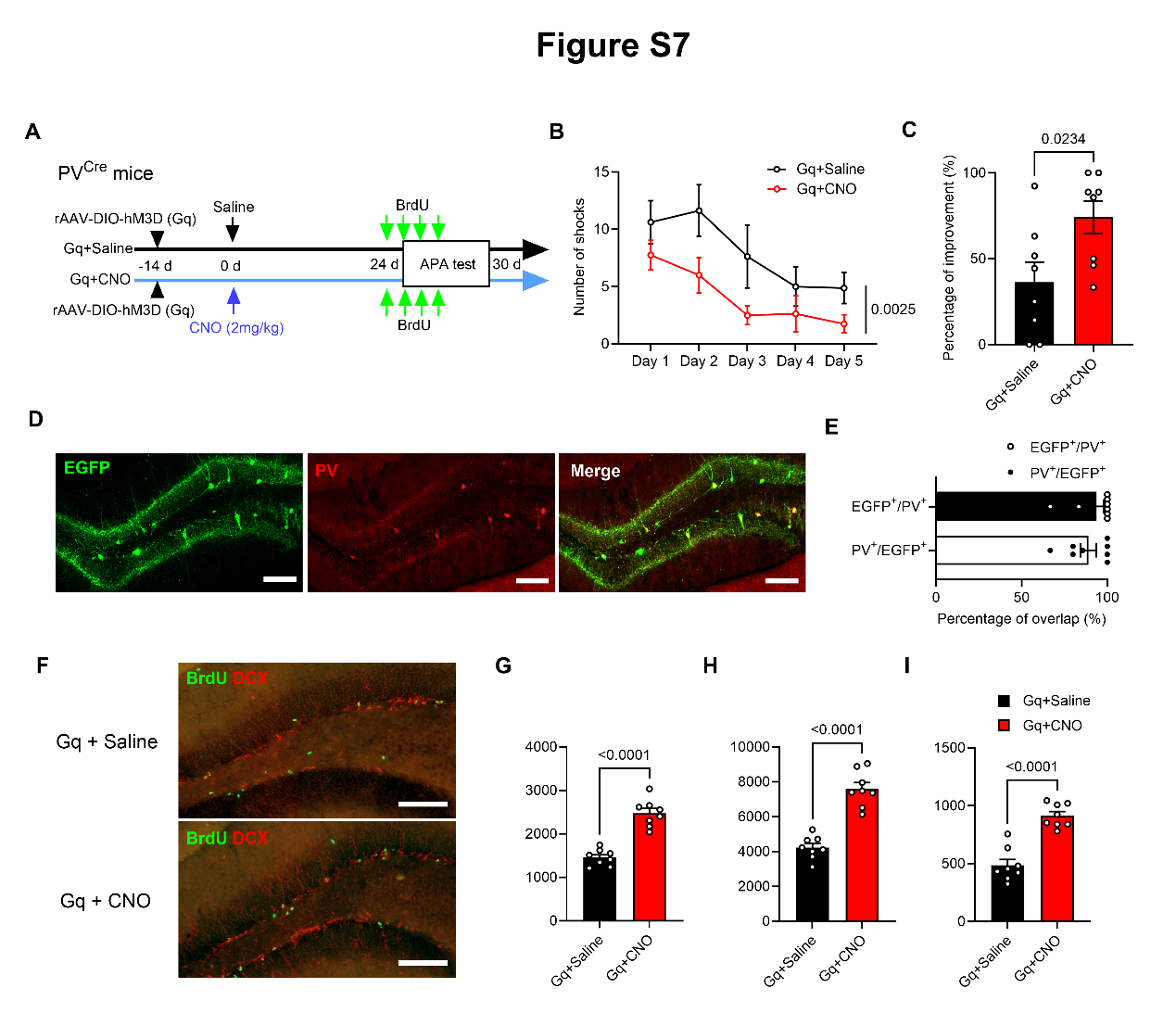


**Figure S7.** Effects of chemogenetic activation of PV interneurons. A) Experimental design showing two groups of PV^Cre^ mice injected with AAV-DIO-hM3D (Gq) virus (Gq+Saline group and Gq+CNO group). B) Quantification of foot shocks received by mice from Gq+Saline group and Gq+CNO group during the APA test [two-way repeated measures (RM) ANOVA with Tukey's post hoc test, F_(1, 14)_ = 13.50, P = 0.0025]. *n* = 8 mice per group. C) Percentage improvement in shock avoidance on the 5th day of the APA test compared to the 1st day [two-tailed unpaired t test with t(14) = 2.543, P = 0.0234]. D) Double immunostaining for Gq-EGFP (green) and PV (red) of the DG. Scale bar, 20 μm. E) Determination of percentages of PV^+^ cells expressing Gq-EGFP (EGFP^+^/PV^+^) and Gq-EGFP^+^ cells expressing PV (PV^+^/EGFP^+^). *n* = 24 brain slices from 8 mice. F) Double immunostaining for BrdU (green) and DCX (red) in the DG of mice from Gq+Saline group and Gq+CNO group. G-I) Quantification of the number of BrdU+ cells (G), DCX+ cells (H), and BrdU+/DCX+ cells (I) in the DG [two-tailed unpaired t test for G: t(14) = 7.913, P < 0.0001; two-tailed unpaired t test for H: t(14) = 7.834, P < 0.0001; and two-tailed unpaired t test for I: t(14) = 6.962, P = 0.0001]. *n* = 8 mice per group.

**
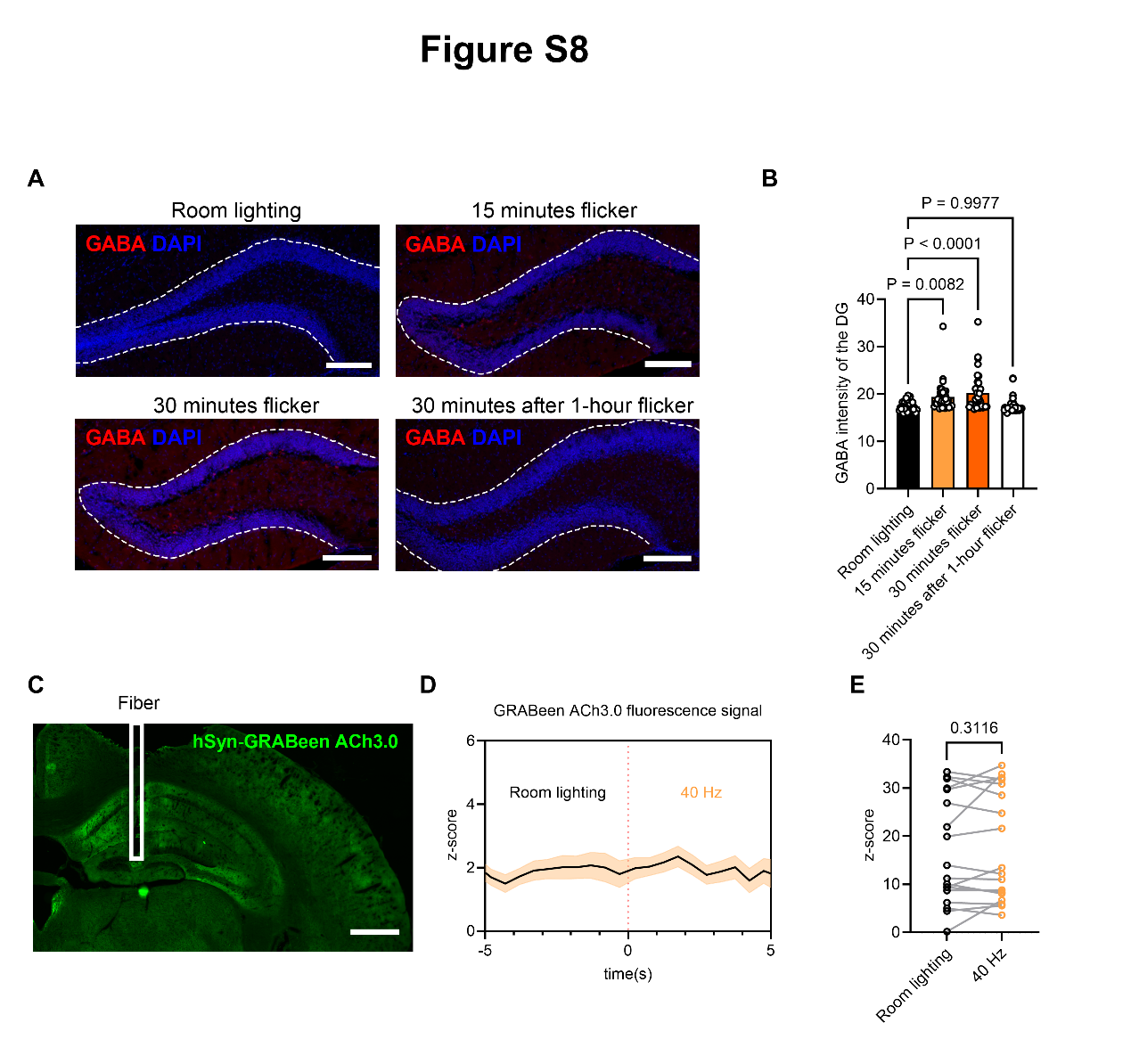
**

**Figure S8.** Determination of altered DG GABA and acetylcholine levels evoked by 40 Hz flicker. A) Immunostaining for GABA (red) with DAPI (blue) of the DG hilus (within the white-colored dotted lines) of different time point groups. These groups are No flicker and 40 Hz light flicker treatment for 15 min (15 minutes flicker), 30 min (30 minutes flicker), and 30 min after 40 Hz light flicker treatment (30 minutes after 1-hour flicker). Scale bars, 100 μm; B) Comparison of GABA intensity among different time points. One-way ANOVA with Tukey's *post hoc* test with P values as indicated. C) An image of the brain coronal section showing recording probe track and GRABeen Ach3.0 fluorescence signal in DG. Scale bars, 200 μm. D) Fiber photometry recording of GRABeen Ach3.0 fluorescence signal showing acetylcholine level in response to 40 Hz light flicker. The average z-score of GRABeen Ach3.0 signals 5 seconds before and during the 40 Hz light flicker treatment was plotted. *n* = 7 mice. E) Quantification of GRABeen Ach3.0 fluorescence intensity 5 seconds before and during 40 Hz light flicker [two-tailed paired t test with t_(21)_ = 0.7886, P = 0.4392].


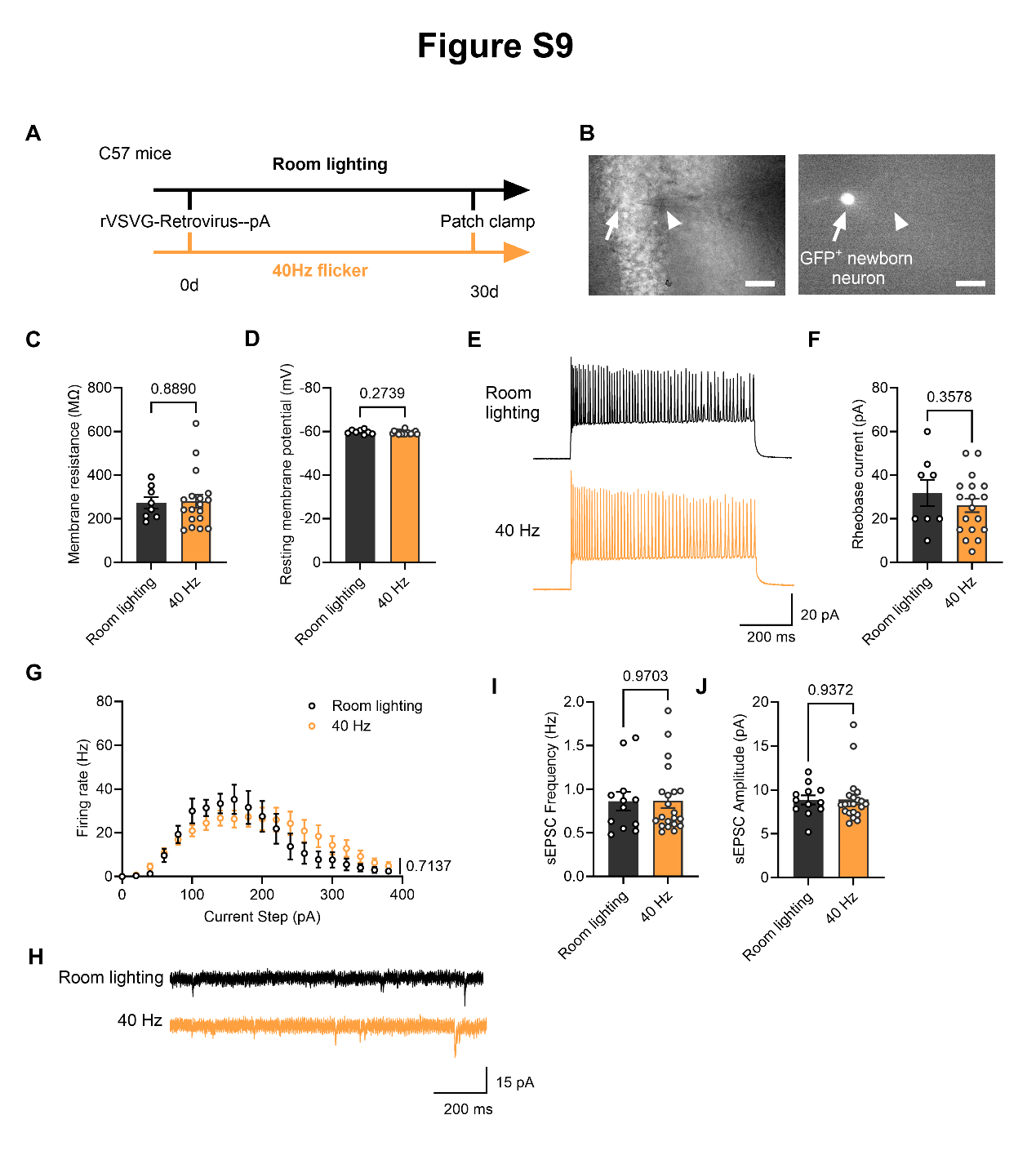


**Figure S9.** Measurements of electrophysiological properties of newborn DCX^+^ cells. A) The experimental design. B) Images of GFP^+^ newborn neurons identified using a fluorescence microscope and phase contrast bright field for patch clamp recording. The arrowheads indicate the glass recording pipette, while the arrows indicate the eGFP-expressing DCX^+^ cell. Scale bars, 20 μm. C) Membrane resistance R_m_ of DCX^+^ cells before and after long-term 40 Hz light flicker treatment [two-tailed paired t test with t_(24)_ = 0.1410, P = 0.8890]. D) Resting membrane potential of DCX^+^ cells before and after long-term 40 Hz light flicker treatment [two-tailed paired t test with t_(24)_ = 1.120, P = 0.2739]. E) Representative firing rate traces recorded in DCX^+^ cells from No flicker and 40 Hz groups. F) The rheobase current recorded in DCX^+^ cells from the No flicker and 40 Hz groups [two-tailed paired t test with t_(24)_ = 0.9375, P = 0.3578]. G) The firing rate recorded in DCX^+^ cells from the No flicker and 40 Hz groups [two-way RM ANOVA with Tukey's *post hoc* test with F_(1, 439)_ = 0.9607, P = 0.3275]. For panels C-G) data were collected from n = 3 mice with 8 cells recorded in the No flicker group, and n = 4 mice with 15 cells recorded in the 40 Hz group. H) Representative sEPSCs traces recorded in DCX^+^ cells from the No flicker and the 40 Hz group. I, J) The recorded frequency (I) and amplitude (J) of sEPSC in DCX^+^ cells from the No flicker and the 40 Hz group. A two-tailed unpaired t test was performed for I [t_(31)_ = 0.03753, P = 0.9703] and J [t_(31)_ = 0.07941, P = 0.9372]. For panels I and J, *n* = 3 mice with 12 cells recorded in the No flicker group, and *n* = 4 mice with 21 cells recorded in the 40 Hz group.


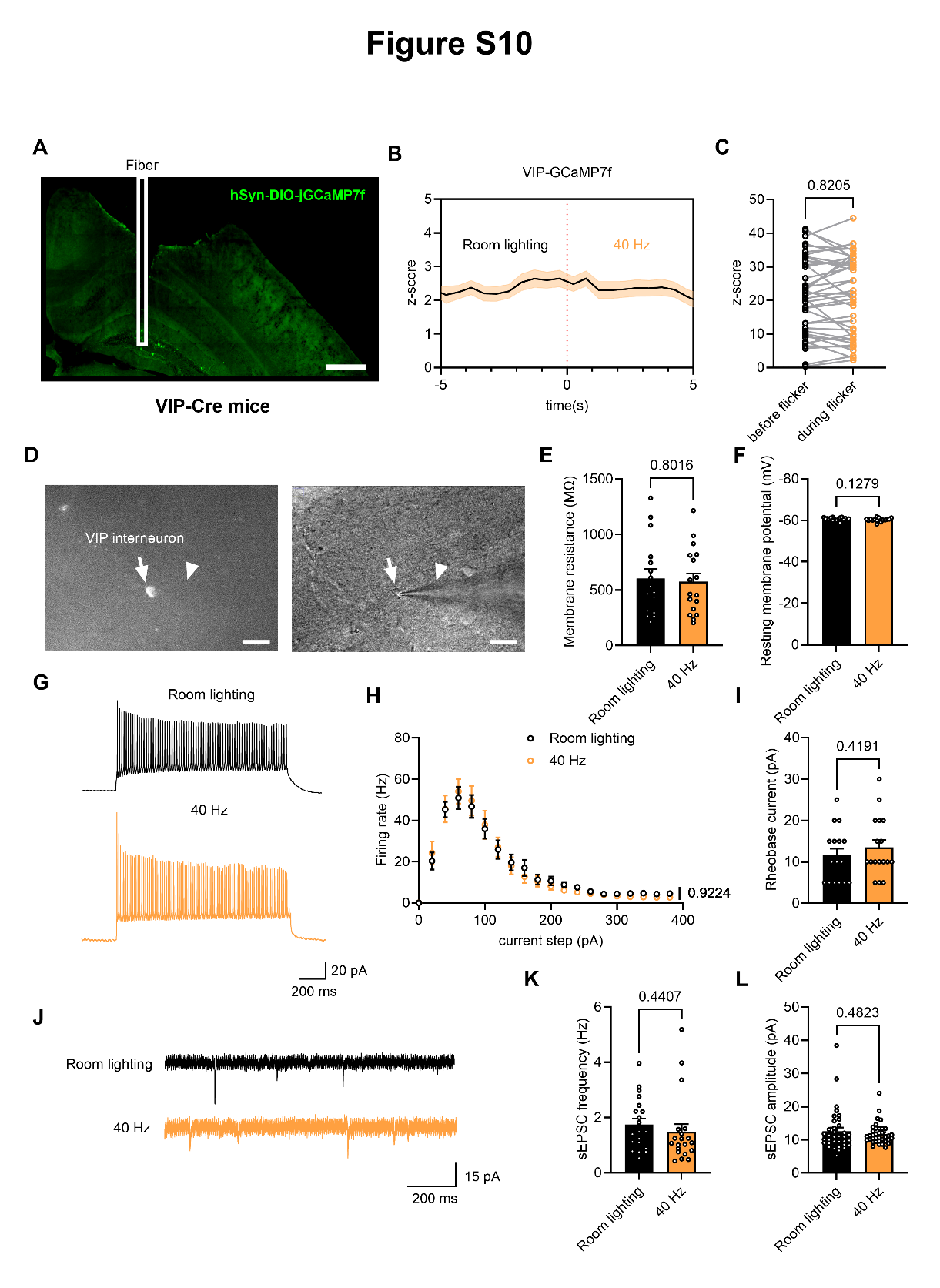


**Figure S10.** Long-term flicker does not affect VIP interneurons in DG. A) An image of the brain coronal section of the left hippocampus showing the recording optic fiber track and the rAAV fluorescence in DG. B) Fiber photometry recording of GCaMP7f signal in VIP interneuron, showing 5 seconds before and during the 40 Hz light flicker treatment. The average z-score of the signals was plotted. *n* = 6 mice. C) Quantification of the GCaMP7f signals of VIP interneurons before and during 40 Hz light flicker [two-tailed paired t test with t_(39)_ = 0.2285, P = 0.8205]. D) Patch clamp recording of VIP interneurons close to the granule layer of the DG. The arrowheads indicate the glass recording pipette, while the arrows indicate VIP interneuron. Scale bars, 20 μm. E) Membrane resistance R_m_ of VIP interneurons before and after long-term 40 Hz light flicker treatment [two-tailed paired t test with t_(31)_ = 0.2534, P = 0.8016]. F) Resting membrane potential of VIP interneurons before and after long-term 40 Hz light flicker treatment [two-tailed paired t test with t_(31)_ = 1.565, P = 0.1279]. G) Representative firing rate traces recorded in VIP interneurons from the No flicker and 40 Hz groups. H) The firing rate recorded in VIP interneurons from the No flicker and 40 Hz groups [two-way RM ANOVA with Tukey's *post hoc* test with F_(1, 620)_ = 0.2363, P = 0.6271]. I) The rheobase current recorded in VIP interneurons from the No flicker and 40 Hz groups [two-tailed paired t test with t_(31)_ = 0.8189, P = 0.4191]. For panels E, F, H, and I, data were collected from *n* = 4 mice with 16 cells recorded in the No flicker group, and *n* = 4 mice with 17 cells recorded in the 40 Hz group. J) Representative sEPSCs traces recorded in VIP interneurons from the No flicker and the 40 Hz group. K, L) The recorded frequency (K) and amplitude (L) of sEPSC in VIP interneurons from the No flicker and the 40 Hz group. A two-tailed unpaired t test was performed for K [t_(38)_ = 0.7792, P = 0.4407] and L [t_(70)_ = 0.7064, P = 0.4823]. For panels K and L, *n* = 4 mice with 20 cells recorded in the No flicker group, and *n* = 4 mice with 20 cells recorded in the 40 Hz group.


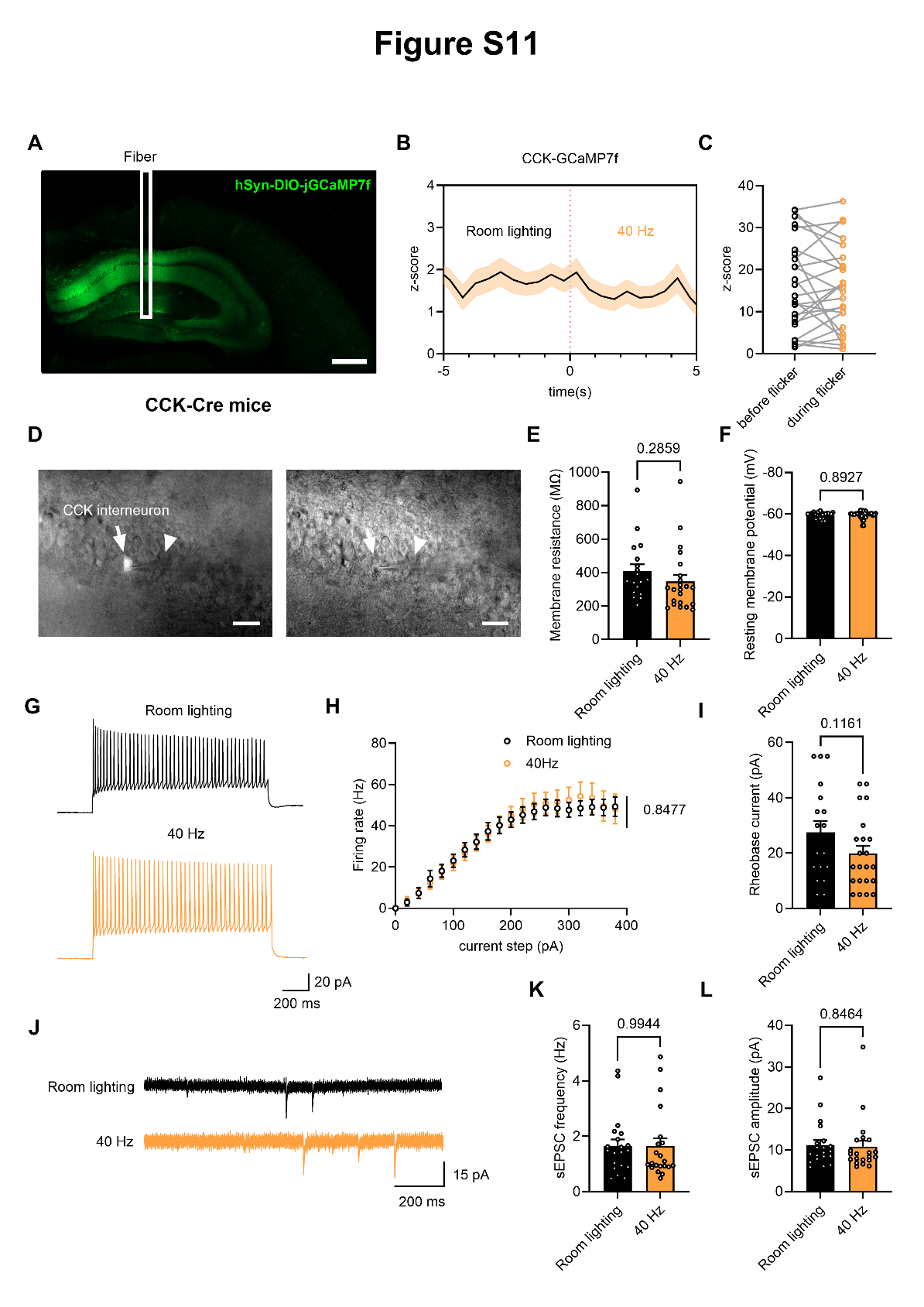


**Figure S11.** Long-term flicker does not affect CCK interneurons in DG. A, An image of the brain coronal section of the left hippocampus showing recording optic fiber track and rAAV fluorescence in DG. B, Fiber photometry recording of GCaMP7f signal in CCK interneuron, showing 5 seconds before and during the 40 Hz light flicker treatment. The average z-score of signals was plotted. *n* = 6 mice. C, Quantification of calcium activity in CCK interneurons before and during 40 Hz light flicker was performed. A two-tailed paired t-test was conducted to assess statistical significance [t_(24)_ = 0.5924, P = 0.5591]. D, Patch clamp recording of CCK interneurons close to the granule layer of the DG. The arrowheads indicate the glass recording pipette, while the arrows indicate the CCK interneuron. Scale bars, 20 μm. E, Membrane resistance R_m_ of CCK interneurons before and during long-term 40 Hz light flicker treatment [two-tailed paired t test with t_(38)_ = 1.082, P = 0.2859]. F, Resting membrane potential of CCK interneurons before and during long-term 40 Hz light flicker treatment [two-tailed paired t test with t_(62)_ = 0.1354, P = 0.8927]. G, Representative firing rate traces recorded in CCK interneurons from the No flicker and 40 Hz groups. H, The firing rate recorded in CCK interneurons from the No flicker and 40 Hz groups [two-way RM ANOVA with Tukey's *post hoc* test with F_(1, 280)_ = 0.09667, P = 0.7561]. I, The rheobase current recorded in CCK interneurons from the No flicker and 40 Hz groups [two-tailed paired t test with t_(38)_ = 1.608, P = 0.1161]. For panels E, F, H, AND I, data were collected from *n* = 5 mice, with 15 cells recorded in the No flicker group, and n = 5 mice, with 19 cells recorded in the 40 Hz group. J, Representative sEPSCs traces recorded in CCK interneurons from the No flicker and the 40 Hz group. K, L, The recorded frequency (K) and amplitude (L) of sEPSC in CCK interneurons from the No flicker and the 40 Hz group. A two-tailed unpaired t test was performed for K [t_(39)_ = 0.007059, P = 0.9944] and L [t_(39)_ = 0.1951, P = 0.8464]. For panels K and L, *n* = 5 mice, with 20 cells recorded in the No flicker group, and *n* = 5 mice, with 21 cells recorded in the 40 Hz group.
